# Speciation Underpinned by Unexpected Molecular Diversity in the Mycorrhizal Fungal Genus *Pisolithus*

**DOI:** 10.1093/molbev/msad045

**Published:** 2023-02-22

**Authors:** Jonathan M Plett, Shingo Miyauchi, Emmanuelle Morin, Krista Plett, Johanna Wong-Bajracharya, Maira de Freitas Pereira, Alan Kuo, Bernard Henrissat, Elodie Drula, Dominika Wojtalewicz, Robert Riley, Jasmyn Pangilinan, William Andreopoulos, Kurt LaButti, Chris Daum, Yuko Yoshinaga, Laure Fauchery, Vivian Ng, Anna Lipzen, Kerrie Barry, Vasanth Singan, Jie Guo, Teresa Lebel, Mauricio Dutra Costa, Igor V Grigoriev, Francis Martin, Ian C Anderson, Annegret Kohler

**Affiliations:** Hawkesbury Institute for the Environment, Western Sydney University, Richmond, Australia; Université de Lorraine, INRAE, Interactions Arbres/Microorganismes, Champenoux, France; Department of Plant-Microbe Interactions, Max Planck Institute for Plant Breeding Research, Köln, Germany; Evolution and Synthetic Biology Unit, Okinawa Institute of Science and Technology Graduate University, Onna, Japan; Université de Lorraine, INRAE, Interactions Arbres/Microorganismes, Champenoux, France; Hawkesbury Institute for the Environment, Western Sydney University, Richmond, Australia; Elizabeth Macarthur Agricultural Institute, NSW Department of Primary Industries, Menangle, Australia; Hawkesbury Institute for the Environment, Western Sydney University, Richmond, Australia; Elizabeth Macarthur Agricultural Institute, NSW Department of Primary Industries, Menangle, Australia; Université de Lorraine, INRAE, Interactions Arbres/Microorganismes, Champenoux, France; US Department of Energy Joint Genome Institute, Lawrence Berkeley National Laboratory, Berkeley, CA; Department of Biological Sciences, King Abdulaziz University, Jeddah, Saudi Arabia; DTU Bioengineering, Technical University of Denmark, Lyngby, Denmark; Architecture et Fonction des Macromolécules Biologiques (AFMB), CNRS, Aix Marseille Université, Marseille, France; INRAE, USC1408 Architecture et Fonction des Macromolécules Biologiques (AFMB), Marseille, France; Hawkesbury Institute for the Environment, Western Sydney University, Richmond, Australia; US Department of Energy Joint Genome Institute, Lawrence Berkeley National Laboratory, Berkeley, CA; US Department of Energy Joint Genome Institute, Lawrence Berkeley National Laboratory, Berkeley, CA; US Department of Energy Joint Genome Institute, Lawrence Berkeley National Laboratory, Berkeley, CA; US Department of Energy Joint Genome Institute, Lawrence Berkeley National Laboratory, Berkeley, CA; US Department of Energy Joint Genome Institute, Lawrence Berkeley National Laboratory, Berkeley, CA; US Department of Energy Joint Genome Institute, Lawrence Berkeley National Laboratory, Berkeley, CA; Université de Lorraine, INRAE, Interactions Arbres/Microorganismes, Champenoux, France; US Department of Energy Joint Genome Institute, Lawrence Berkeley National Laboratory, Berkeley, CA; US Department of Energy Joint Genome Institute, Lawrence Berkeley National Laboratory, Berkeley, CA; US Department of Energy Joint Genome Institute, Lawrence Berkeley National Laboratory, Berkeley, CA; US Department of Energy Joint Genome Institute, Lawrence Berkeley National Laboratory, Berkeley, CA; US Department of Energy Joint Genome Institute, Lawrence Berkeley National Laboratory, Berkeley, CA; Botanic Gardens and State Herbarium, Department for Environment and Water, Adelaide, Australia; Department of Microbiology, Universidade Federal de Viçosa, Viçosa, Brazil; Laboratório de Associações Micorrízicas, Instituto de Biotecnologia Aplicada à Agropecuária (BIOAGRO), Av. P. H. Rolfs, s/n, Campus UFV. Bolsista Pesquisador do Conselho Nacional de Desenvolvimento Científico e Tecnológico (CNPq), Brasília, DF, Brazil, Viçosa, Brazil; US Department of Energy Joint Genome Institute, Lawrence Berkeley National Laboratory, Berkeley, CA; Plant and Microbial Biology Department, University of California Berkeley, Berkeley, CA; Université de Lorraine, INRAE, Interactions Arbres/Microorganismes, Champenoux, France; Hawkesbury Institute for the Environment, Western Sydney University, Richmond, Australia; Université de Lorraine, INRAE, Interactions Arbres/Microorganismes, Champenoux, France

**Keywords:** mycorrhizal symbiosis, host specificity, trehalose, CAZyme, transposable elements, effector, evolution

## Abstract

The mutualistic ectomycorrhizal (ECM) fungal genus *Pisolithus* comprises 19 species defined to date which colonize the roots of >50 hosts worldwide suggesting that substantial genomic and functional evolution occurred during speciation. To better understand this intra-genus variation, we undertook a comparative multi-omic study of nine *Pisolithus* species sampled from North America, South America, Asia, and Australasia. We found that there was a small core set of genes common to all species (13%), and that these genes were more likely to be significantly regulated during symbiosis with a host than accessory or species-specific genes. Thus, the genetic “toolbox” foundational to the symbiotic lifestyle in this genus is small. Transposable elements were located significantly closer to gene classes including effector-like small secreted proteins (SSPs). Poorly conserved SSPs were more likely to be induced by symbiosis, suggesting that they may be a class of protein that tune host specificity. The *Pisolithus* gene repertoire is characterized by divergent CAZyme profiles when compared with other fungi, both symbiotic and saprotrophic. This was driven by differences in enzymes associated with symbiotic sugar processing, although metabolomic analysis suggest that neither copy number nor expression of these genes is sufficient to predict sugar capture from a host plant or its metabolism in fungal hyphae. Our results demonstrate that intra-genus genomic and functional diversity within ECM fungi is greater than previously thought, underlining the importance of continued comparative studies within the fungal tree of life to refine our focus on pathways and evolutionary processes foundational to this symbiotic lifestyle.

## Introduction

The majority of terrestrial plants exist naturally in symbiosis with mycorrhizal fungi; a form of mutualistic interaction that enhances the ecological fitness of individual plants and shapes the structure and dynamics of plant populations and communities (van der Heijden et al. 2015; Bennett et al. 2017; Tedersoo et al. 2020). Within forest ecosystems, due to low nutrient availability, most trees have evolved beneficial mutualistic associations with ectomycorrhizal (ECM) fungi, leading to approximately one-third of soil microbial biomass in forest ecosystems being made up of ECM hyphae (Högberg and Högberg 2002; Ekblad et al. 2013). Within this mutualism, the fungal partner can receive up to 20–30% of photosynthetically derived carbon from their tree hosts in return for growth limiting nutrients, such as nitrogen and phosphorus (Ouimette et al. 2020). Due to their fundamental roles in nutrient cycling, these mutualistic relationships are thought to have shaped forest ecosystems since their inception (Martin et al. 2017 ; Strullu-Derrien et al. 2018; Anthony et al. 2022). The efficient exchange of nutrients between the two partners requires the development of a structure called the “Hartig net”—the ingrowth of ECM hyphae into the intercellular space of the root such that the interface between fungal and plant cells are maximized. The development of this Hartig net requires the modulation of both host and fungal transcriptomes and signaling pathways, which have been described in a number of distantly related ECM lineages (Johansson et al. 2004; Wright et al. 2005; Kohler et al. 2015; Plett, Tisserant et al. 2015; Daguerre et al. 2016; Liao et al. 2016; Basso et al. 2020; Bouffaud et al. 2020; Paparokidou et al. 2021). The understanding of which pathways are functionally conserved across closely related ECM fungal taxa, however, is still under-explored.

As opposed to obligate mutualistic symbionts, like arbuscular mycorrhizal fungi, ECM fungi are thought to have evolved 72–82 times from an ancestral saprotrophic form (Martin et al. 2016; Tedersoo and Smith 2017; Brundrett and Tedersoo 2018; Sanchez-Garcia et al. 2020). A number of recent studies have compared ECM fungal genomes with their ancestral saprotrophic lineages including brown- and white-rot fungi (Kohler et al. 2015; Hess et al. 2018; Murat et al. 2018; Sato and Toju 2019; Miyauchi, Kiss et al. 2020; Lebreton et al. 2021). Common to these analyses are the demonstration that ECM fungal lineages have a restricted complement of CAZymes, a proliferation of transposable elements (TEs), and the evolution of lineage-specific genes such as small secreted proteins (SSPs). The order Boletales is among the most species-rich orders in the Agaricomycetes, with species in the suborders Boletineae, Sclerodermatineae, and Suillineae known to establish mutualistic associations with diverse host plants from woodland environments (Bougher 1995; Henkel et al. 2002; den Bakker et al. 2004; Sato et al. 2007; Hosen et al. 2013; Wu et al. 2016; Wu et al. 2022). Sharing a single origin of symbiosis from a brown-rot ancestor (Krah et al. 2018), ECM lineages within the Boletales are of interest from both an evolutionary as well as an ecological perspective due to accelerated rates of speciation and host expansion (i.e., number of host species; Wu et al. 2016; Sato and Toju 2019), and due to their importance as both early- and late-stage successors in natural ecosystems (Ortega-Martínez et al. 2011). Therefore, focusing on the genomic and functional aspects of speciation within a single genus of this order will afford a richer understanding of both the evolutionary pathways following the switch from saprotrophy to mutualistic symbiosis as well as to understanding ecosystem services provided by ECM fungi including the metabolism and retention of carbon sourced from a host plant.

Within the Boletales, the ECM genus *Pisolithus* has the potential to be a versatile model to probe this area of research as the generus is both globally dominant and, due to colonizing both gymnosperm and angiosperm hosts, gives the opportunity to explore what pathways have developed to support host colonization. Further, as a strong colonizer of host roots (Cairney and Chambers 1997; Plett, Kohler et al. 2015), and due to their extensive production of long-range exploratory hyphae within soil (Agerer 2001), the genus *Pisolithus* can also serve as a model to understand the dynamics underpinning the ability of ECM fungi to obtain and sequester carbon from host sources (Hortal et al. 2017; Plett, Singan et al. 2020). A recent intra-order genomic analysis of the Boletales found that *Pisolithus* and *Scleroderma* are unique in encoding an extremely low content of CAZymes (Wu et al. 2021), a factor that would make these genera especially reliant on host carbon as they lack the necessary enzymes to obtain this nutrient from dead organisms. Over the last two centuries, the natural geographic range has expanded due to the use of *Pisolithus* as inoculum in bioenergy and forestry plantations. *Pisolithus microcarpus*, originating from Australasia, has spread to Africa, North and South America, and Europe in eucalypt plantations, whereas *P. tinctorius* sensu stricto (s.str.), originally from the Northern hemisphere, can be found in pine plantations in a number of countries in the Southern hemisphere. The genomes of these two species have been previously published and found to have a range of differences in assembly size and in gene content (Kohler et al. 2015). However, as these two species have different host ranges, and because there are currently 19 described species (Martin et al. 2002; Phosri et al. 2012; Lebel et al. 2018), it is unknown how broadly applicable these findings are across the genus.

To address these gaps in our knowledge, we sequenced the genomes of seven additional *Pisolithus* species, and generated improved draft genomes of *P. microcarpus* and *P. tinctorius* s.str., to understand the degree of genomic conservation between these nine genomes. This study presents key foundational information for understanding symbiosis development and functioning within a globally dominant genus and generate the resources to better understand ECM fungal-host specificity.

## Results

### The *Pisolithus* Genus Encodes a Small Core genome

**Fig. 1.**
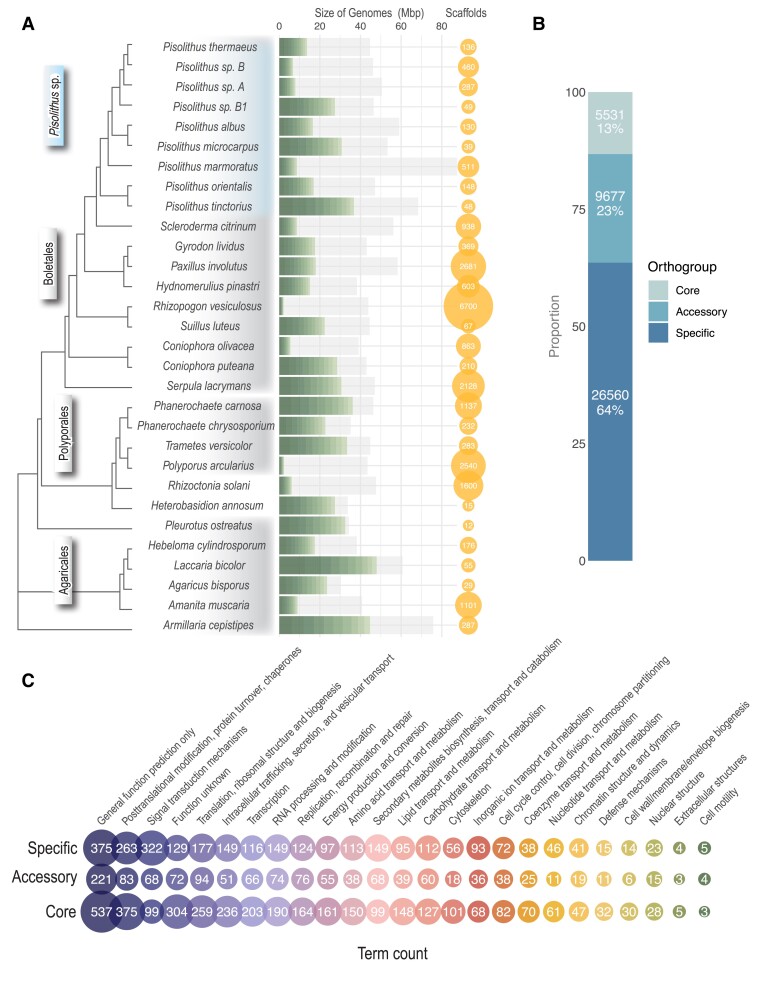
General characteristics of *Pisolithus* genomes. (*A*) Multi-locus phylogeny of all fungal genomes utilized in this study (642 single-copy orthologous genes were used for the tree construction). Green gradient bars show the size of ten largest scaffolds, and grey shadows represent the total size of genomes. The bubbles beside the graph indicate the number of scaffolds in the genome assemblies. (*B*) When all *Pisolithus* genomes are compared, they can be subdivided into three categories: (*i*) the core genome (the set of genes shared by all genomes), (*ii*) the accessory genome (ACC.; the set of genes present in some but not all genomes), and (*iii*) the specific genome (SPEC; genes that are unique to a single genome). (*C*) The count of gene functions by KOG classification for the core, accessory, and specific genome.

Phylogenetic reconstruction of the nine sequenced *Pisolithus* species indicated that *P. tinctorius* s.str. isolate Marx270, hosted by gymnosperms, Fagaceae (*Quercus*) and Cistaceae (*Cistus*), and *P. orientalis* isolate OTSU, a known symbiont of coniferous hosts, were the most basal species of the genus sequenced and the most closely related to *Scleroderma citrinum* (fig. 1*a*). When the primary and secondary scaffolds were accounted for, the genome sizes of these nine species, with the exception of *P. marmoratus* isolate 16C, tended to be similar to the other ecological groups of fungi considered (fig. 1*a*; supplementary fig. S1*a*and file S1, Supplementary Material online). When pairs of allelic scaffolds are identified by similarity on the nt level using blat (> 50% coverage and > 90% identity), the primary scaffold is defined as the longer of the two scaffolds. Using only the primary scaffolds, we visualized the number and size of scaffolds compared with the genome size for *Pisolithus* genomes and found that all species showed a fragmented genome as none had scaffold 1–10 covering more than 60% of the genome size (fig. 1*a*). We assessed and confirmed that genome completeness for *Pisolithus* species was above 95%, suggesting the genome assemblies covered nearly all fungal common genes (see BUSCO in supplementary fig. S1*b*and file S2, Supplementary Material online). The *Pisolithus* genomes were found to have much higher TE content (up to 60%) when compared with the other fungi in our analysis, whereas the number of genes was comparable across all fungal lifestyles (supplementary figs. S1*b*, S2 and files S2 and S3, Supplementary Material online). The TE coding space was found to be a major driver separating *Pisolithus* and other ECM genomes from white and brown rot fungi and pathogens (supplementary fig. S3*a*, Supplementary Material online). Of the TEs found within the primary assembly, the majority were found to be from the long terminal repeat retrotransposons, DNA transposons, and unclassified repeats (supplementary fig. S2; S3*b*and file S3, Supplementary Material online).

Conservation of predicted genes was low across the *Pisolithus* species considered. When the genes were clustered based on homology, hereafter referred to as gene orthogroups, we found that 5,531 orthogroups (13% of all orthogroups) were considered to be part of the “core” genome (containing a minimum of one ortholog in all genomes sequenced), 9,677 were “accessory” (containing orthologs in a minimum of two genomes; 23% of total), and 26,560 were species-specific (63%; fig. 1*b*; table 1). In all three classes, we found that there was a much larger portion of genes that could not be assigned to a KOG functional group in the accessory and specific gene orthogroups when compared with the core genome (87% and 89% vs. 35%, respectively; fig. 1*c*; supplementary file S4, Supplementary Material online). Within the core genome, the most highly represented KOG functions were “posttranslational modification and protein turnover”, “translation”, and “intracellular trafficking”. Although KOG functional classifications were relatively equally represented (by percentage of orthogroups; supplementary file S4, Supplementary Material online) across the three class groupings, the annotations of genes within these categories differed between the core and specific genomes. For example, within “carbohydrate metabolism” the core genome predominantly contained orthogroups associated with transporters and the biosynthesis of sugars in the fructose/glucose/glycerol pathway, whereas the specific genome contained orthogroups involved in trehalose synthesis and catabolism (33% of gene orthogroups). Orthogroups accounting for secondary metabolism as well as cellular perception and communication were among the few KOG classifications that exhibited a greater percentage of annotated gene orthogroups in the specific genomes (fig. 1*c*; supplementary file S4, Supplementary Material online).

**Table 1.** *Pisolithus* Genes Differentially Expressed During Colonization of a Receptive Host. *Pinus Sylvestris* was used as the Host for *P. tinctorius* s.str., *Eucalyptus globulus* for *P. microcarpus* 441, whereas *Eucalyptus grandis* was used for the remaining fungi. Acc, accessory; Spec, specific. For each interaction, the number of differentially expressed (up- or down-regulated during colonization; log2 Fold change > 1, FDR-pvalue < 0.05) SSPs is given and how many of the gene belongs to the core, accessory (Acc) or species-specific (Spec) gene set (fast-ortho clustering).

	ECM Up	Core	Acc	Spec	% Spec	ECM Down	Core	Acc	Spec	% Spec	Genome Spec	Total	% Spec
*Pisolithus* sp. B	750	452	256	42	5.6	725	467	226	32	4.4	1,012	12,614	8.0
*P. albus*	352	197	102	53	15.1	223	138	45	40	17.9	2,417	11,461	21.1
*P. microcarpus*	179	106	52	21	11.7	36	23	11	2	5.6	2,550	11,542	22.1
*P. marmoratus*	324	201	68	55	17.0	344	152	57	135	39.2	5,575	15,639	35.6
*P. tinctorius*	534	318	131	85	15.9	230	122	62	46	20.0	2,839	12,084	23.5

### Genes Belonging to the Core Genome of *Pisolithus* are more Likely to be Induced by Symbiosis

**Fig. 2.**
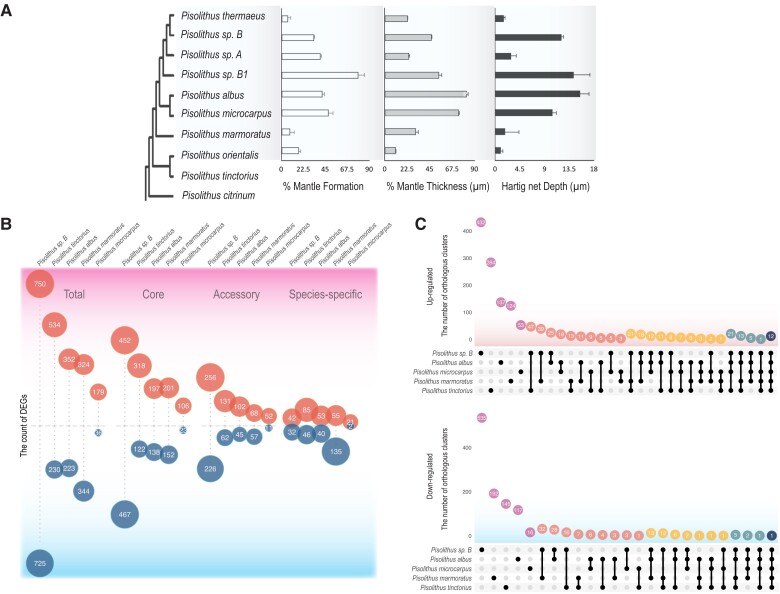
*Pisolithus* species exhibit phylogenetically linked host colonization abilities and gene expression patterns. (*A*) Percent of lateral roots of *E. grandis* displaying mantle formation across eight species of *Pisolithus,* the measured average thickness of these mantles, and the average measures depth of Hartig net penetration into root apoplastic space. + SE; (*B*) The count of differentially expressed genes (> 1 log2 FC; FDR adj. *P* < 0.05) in orthogroups during host colonization; (*C*) Upset plot showing the number of orthogroups containing up- or down-regulated genes (> 1 log2 FC; FDR adj. *P* < 0.05) during host root colonization.

Generally, the most basal *Pisolithus* species considered in this study (e.g., *P. tinctorius* s.str.) are known to colonize gymnosperms, Fagaceae and Cistaceae, whereas the remaining species typically colonize angiosperm hosts, suggesting a host shift during the evolution of these core genes. We tested the ability of the *Pisolithus* species to colonize the model tree *E. grandis* (fig. 2*a*). Colonization ability was found to reflect phylogenetic relationships, whereby species more closely related to *P. tinctorius* s.str. (i.e., *P. orientalis*, *P. marmoratus*) colonized *E. grandis* poorly (<20% lateral roots exhibiting mantle formation; fig. 2*a*), whereas isolates more closely related to *P. microcarpus* (i.e., *P. albus*, *Pisolithus* sp. B, *Pisolithus* sp. C) colonized between 40% and 80% of *E. grandis* lateral roots. *P. thermaeus*, isolated from geothermal soils dominated by the shrub *Leptospermum tenuicaulis* and *Leptopsermum scoparium*, was also unable to form a Hartig net or mantle around the lateral roots of *E. grandis* (i.e., 6.9%; fig. 2*a*), even though both of these hosts are from the Myrtaceae.

In order to identify the fungal genes differentially regulated in colonized root tissues, we sequenced the transcriptomes of five *Pisolithus* species when colonizing a compatible host (table 1; supplementary tables S5–S10, Supplementary Material online). When we considered whether the identified significantly differentially expressed genes (DEGs; FDR adjusted *P* < 0.05) belonged to the core, accessory, or specific genomes of each species as classified using all nine genomes (fig. 1*b*), we found that the majority of DEGs (53–64%) in all species tested belonged to the core genome of the *Pisolithus*. Regulation of species-specific genes, meanwhile, ranged between 5% (*Pisolithus* sp. B) and 28% (*P. marmoratus*; table 1). Surprisingly, given the large percentage of core DEGs, only 12 core orthologous functional orthogroups (0.8% of all orthogroups; fig. 2*c*) were significantly induced during host colonization between all species and one core cluster was repressed in colonized tissues (0.09% of all orthogroups; fig. 2*c*; supplementary files S6–S10, Supplementary Material online). Although there were 48 gene orthogroups shared between DEGs of four of five species interactions, the vast majority of gene orthogroups were species-specific (fig. 2*b* and *c*; supplementary files S6–S10, Supplementary Material online).

### SSPs are Significantly Closer to TEs but Smaller Accessory SSPs are Induced by Symbiosis

**Fig. 3.**
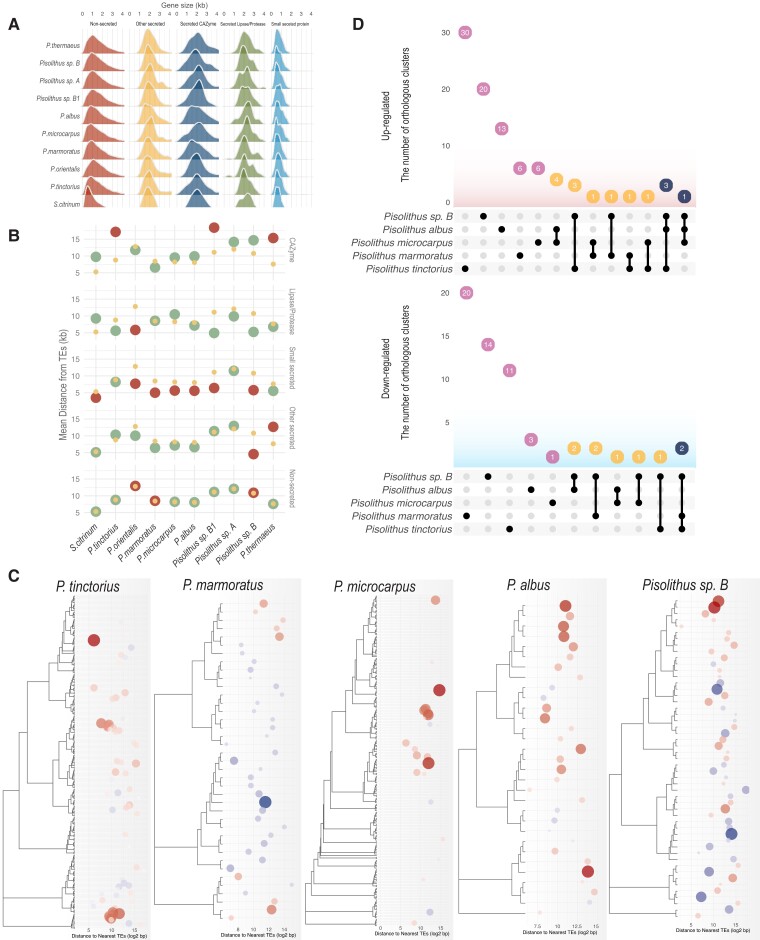
*Pisolithus* SSPs are significantly closer to TE/repeat rich regions of the genome although this does not predict symbiosis induction patterns. (*A*) Gene size in kilobase for five major gene classes in all sequenced *Pisolithus* sp.; (*B*) Mean distances between repeat elements and genes. Small yellow dots: Mean distances of the 10,000 randomly reshuffled genome models (to generate a null hypothesis). Green circles: Mean distances observed in the genomes with no statistical significance (*P* > 0.05). Red circles: Mean distances observed in the genomes with statistical significance (*P* < 0.05). (*C*) Heatmap of significantly regulated SSPs in five *Pisolithus* species, whereby blue coloration indicates gene repression, red coloration indicates induction of transcription. Intensity of coloration indicates relative repression/induction, respectively. SSPs are clustered according to the sequence similarity. Circle size corresponds to absolute values in differential expression. The location of circles indicates TE-gene distance in log2 bases shown in the X axis. (*D*) Upset plot comparing core and accessory gene orthogroups with higher transcript abundance in five different *Pisolithus* species colonizing a host root (top panel) and with lower transcript abundance (bottom panel).

Across the entire genomic space, proximity of coding genes to repeat elements was calculated (supplementary fig. S4, Supplementary Material online). The distance of genes to nearest repeat elements within 4.5 kb was defined as “close proximity” because coding genes tend to be smaller than 4 kb (fig. 3*a*). Of five broad categories including non-secreted proteins (i.e., intracellular proteins), secreted proteins, SSPs, lipases/proteases, and CAZymes, we observed that the genomic location of genes coding for SSPs were more frequently found to be significantly closer to nearest repeat element than other gene classifications (fig. 3*b*; *P* < 0.05). The genome content of non-LTR retrotransposons varied significantly with the gene count of SSPs, suggesting potential association between two features (R2 = 0.18; *P* < 0.05; PERMANOVA; supplementary fig. S5*a*and file S11, Supplementary Material online). The most common genes found close to TE/repeats were genes encoding for PFAMs associated with zinc finger domain-containing proteins, and heterokaryon incompatibility-related proteins (supplementary fig. S5*b*and file S11, Supplementary Material online).

As the association of the SSPs with repeat elements and TEs may drive evolution of these sequences as well as their transcriptional profiles, we analyzed the regulation of SSPs during symbiosis with a host in relation to their proximity to TEs. Interestingly, a similar number of SSPs was expressed regardless of species considered or the growth conditions (table 2). In the five species considered, we found no clear pattern between gene-to-TE distance and the likelihood of those genes being differentially regulated (fig. 3*c*; supplementary fig. S6, Supplementary Material online). Sequence similarity (fig. 3*c*), and SSP size (supplementary fig. S7, Supplementary Material online) were related to observed SSP expression. A further study of those SSPs significantly regulated during colonization found that, with the exception of *Pisolithus* sp. B, between 65% and 75% came from the accessory/core genome (table 3). This would suggest that there may be SSP orthologs similarly regulated by colonization between the *Pisolithus* species studied. This was not the case, with zero differentially regulated SSP orthogroups being similarly induced or repressed by colonization when we compared all species together (fig. 3*d*). The largest overlap in the fold change in the expression of SSP orthologs was found between *P. microcarpus* and *P. albus*, with 45% of SSPs being commonly regulated by host presence. This overlap, appeared to decrease with phylogenetic distance (fig. 3*d*). To ascertain if this same pattern was observed when we only considered normalized expression of the SSPs under axenic conditions and when colonizing a host, we plotted the expression of all accessory and core SSPs that were maintained in >2 of the species considered (supplementary fig. S8, Supplementary Material online). This analysis demonstrated a far greater similarity between the species (supplementary fig. S8*b*, Supplementary Material online) with 67–70% of the 129 SSPs found to be highly, but similarly, expressed between free-living mycelium (FLM) and ECM, suggesting that the large majority of *Pisolithus* SSPs are constitutively expressed and that only a small number have evolved to be responsive to the presence of a host root.

**Table 2.** *Pisolithus* Small Secreted Protein (SSP) Genes Expressed During Colonization of a Receptive Host. *Pinus sylvestris* was used as the host for *P. tinctorius* s.str., *Eucalyptus globulus* for *P. microcarpus* 441, whereas *Eucalyptus grandis* was used for the remaining fungi. Acc, accessory; Spec, specific. For each interaction the number of expressed SSPs is given and how many of the gene belongs to the core, accessory (Acc) or species-specific (Spec) gene set (fast-ortho clustering). The SSP annotation and expression was taken from supplementary tables S6–S10, Supplementary Material online.

	# SSP expressed	Core	Acc	Spec	% Spec
*Pisolithus* sp. B	242	57	131	54	22
*P. albus*	203	50	62	91	45
*P. microcarpus*	213	56	75	82	38
*P. marmoratus*	400	60	57	283	71
*P. tinctorius*	273	46	84	143	52

**Table 3.** *Pisolithus* Small Secreted Protein (SSP) Genes Differentially Expressed During Colonization of a Receptive Host. *Pinus sylvestris* was used as the host for *P. tinctorius* s.str., *Eucalyptus globulus* for *P. microcarpus* 441, whereas *Eucalyptus grandis* was used for the remaining fungi. Acc, accessory; Spec, specific. For each interaction, the number of differentially expressed (up- or down-regulated during colonization; log2 Fold change > 1, FDR-pvalue < 0.05) SSPs is given and how many of the gene belongs to the core, accessory (Acc) or species-specific (Spec) gene set (fast-ortho clustering). The SSP annotation and expression was taken from supplementary tables S6–S10, Supplementary Material online.

	# SSP	% of expressed SSPs	% of ECM Up-regulated Genes	Core	Acc	Spec	% spec Up-regulated SSPs	#	% of expressed SSPs	% of ECM Down-regulated Genes	Core	Acc	Spec	% Spec Down-regulated SSPs
	Up							SSP						
								Down						
*Pisolithus* sp. B	29	12	4	11	17	1	3	22	9	3	10	12	0	0
*P. albus*	24	12	7	3	12	9	38	7	3	3	3	3	1	14
*P. microcarpus*	13	6	7	0	10	3	23	3	1	8	1	1	1	33
*P. marmoratus*	9	2	3	4	2	3	33	30	8	9	4	8	18	60
*P. tinctorius*	43	16	8	9	19	15	35	15	5	7	3	9	3	20

### *Pisolithus* Exhibits Unique Gene Expansions in Enzymes Associated With Symbiotic Sugar metabolism

**Fig. 4.**
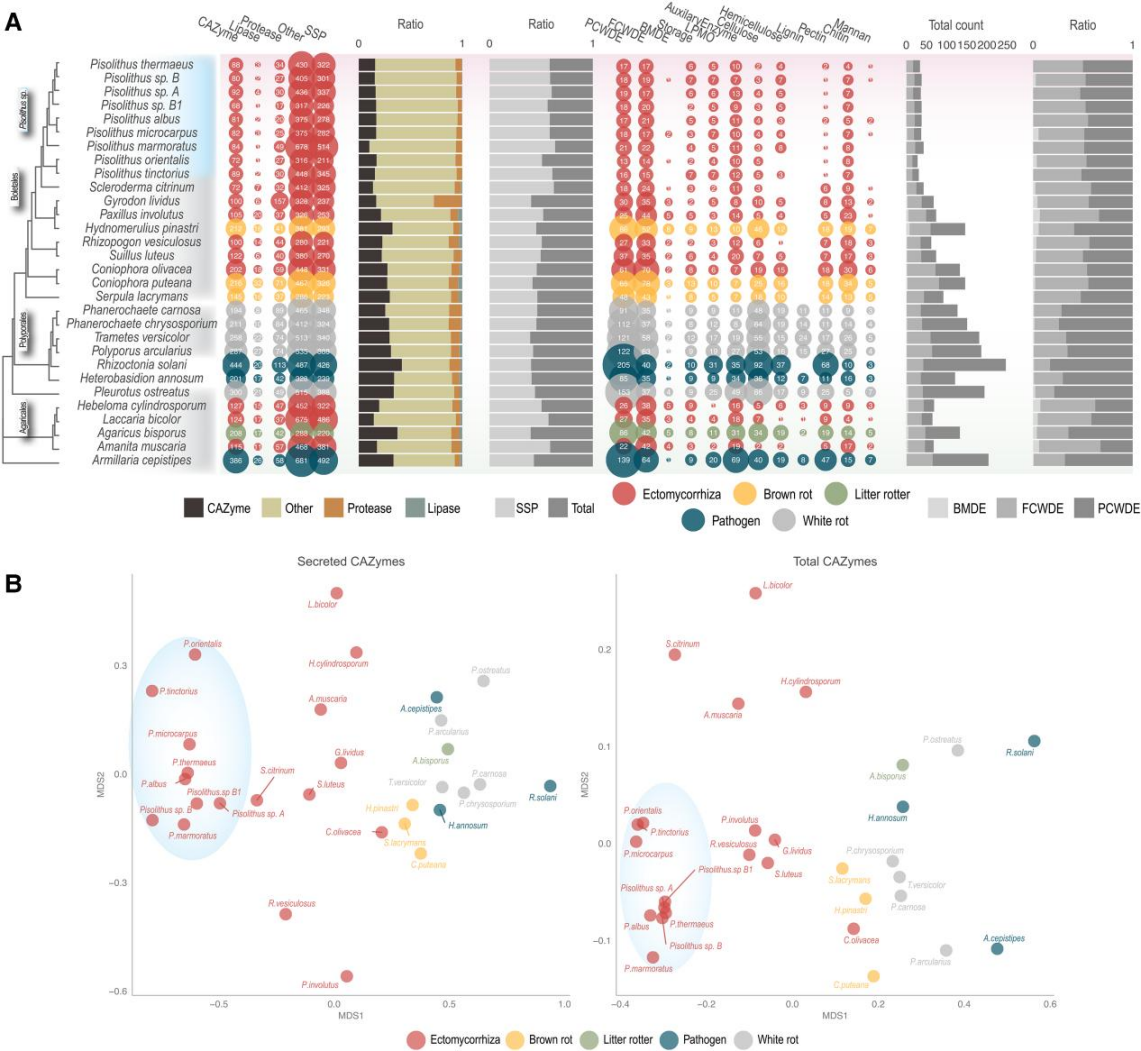
Predicted secretome of *Pisolithus* are distinct from other fungal lifestyles. (*A*) The theoretical secretomic profiles of 30 fungi across four fungal lifestyles. The first bubble plot on the left shows the number of secreted genes for CAZymes, lipases, proteases, and others (i.e., all secreted proteins not in these first three groups). The SSP group is a subcategory showing the number of small secreted proteins (< 300 aa). The size of bubbles corresponds to the number of genes, fungi are colored according to their ecology. The first bar plots (in the middle) represent the ratio of CAZymes, lipases, proteases, to all secreted proteins (left); and the ratio of SSPs among the entire secretome (right). The second bubble plot on the right side of the panel shows CAZymes grouped according to their functions including plant cell wall degrading enzymes (PCWDE) and fungal-cell wall degrading enzymes (FCWDE), peptidoglycan (i.e., bacterial membrane) degrading enzymes (BMDE), trehalose, starch, glycogen degrading enzymes (Storage), lytic polysaccharide monooxygenase (LPMO), substrate-specific enzymes for cellulose, hemicellulose, lignin, and pectin (plant cell walls); chitin, glucan, mannan (fungal-cell walls). The second bar plots (far right) show the total count of genes including PCWDE, MCWDE, and BMDE (left); and the proportion of PCWDE, MCWDE, and BMDE (right). (*B*) CAZyme profiles separate based on genus and fungal lifestyle. Genomes were grouped by secreted CAZyme domains (left column) and total CAZyme domains in genomes (right column). The fungi are grouped according to the ecology and labeled with their full species name. *Pisolithus* genomes are encircled in a blue oval.

Given the dependency of ECM fungi on secreted hydrolytic enzymes for both entering into symbiosis with their hosts and for nutrient acquisition, we analyzed the profiles of CAZymes, proteases, and lipases from the *Pisolithus* species sequenced when compared with 25 fungi across the saprobic-mutualistic continuum (fig. 4*a*). Although the number of enzymes associated with degradation of more complex sugars was comparable between all fungi considered, CAZymes associated with plant- and fungal-cell wall degradation were restricted in number when compared with saprotrophic fungi with approximately 21% of variance in secreted CAZymes explained by fungal ecology (supplementary fig. S5*a*, Supplementary Material online; *P* < 0.05; PERMANOVA; supplementary file S11, Supplementary Material online). When the different genomes were compared based on the identity of their secreted and non-secreted CAZyme profile, it was found that all *Pisolithus* species formed a distinct group, separate from all other fungal genomes considered (fig. 4*b*). This separation was due both to the appearance/decay of certain CAZymes as well as the unique expansions of specific secreted/total CAZyme families among *Pisolithus* (supplementary fig. S9 and file S12, Supplementary Material online). Among those CAZyme families showing expansion, we found several enzymes associated with trehalose, including α, α-trehalase (GH37), which breaks down trehalose into glucose, and the α, α-trehalose-phosphate synthase family (glycosyltransferase family GT20) which includes trehalose synthase 1 (TPS1) and trehalose synthase 2 (TPS2). Across the genomes considered, fungal ecology, non-LTR retrotransposons, and unclassified repeats seemed to be associated with GTs as 15% of variation in GTs was significantly explained by these features (supplementary fig. S5*a*, Supplementary Material online; *P* < 0.05; PERMANOVA; supplementary file S11, Supplementary Material online). Among GT-encoding genes, there was a tendency of GT20 being close to repeat elements (supplementary fig. S10, Supplementary Material online). The genome coverage of TEs was positively correlated with the number of selected GTs including GT20 (R = 0.45–0.85; *P* < 0.01; supplementary fig. S11, Supplementary Material online). The mycorrhizal lifestyle tended to show high TE coverage with a large number of the GTs (supplementary fig. S11, Supplementary Material online). Particularly, *Pisolithus* species were significantly different from the rest of the fungi in terms of the gene count of GT20 and GT90 and the TE coverage in the genomes (Kruskal–Wallis test, *P* < 0.05; ANOVA-TuckeyHSD, *P* < 0.05; supplementary file S13, Supplementary Material online).Within the Pisolithaceae, the expression of GTs was fairly positively correlated with their relative proximity to repeat elements. GT20 showed a clear positive correlation with significance (R = 0.18–0.72; *P* < 0.05) except for *P. tinctorius* s.str with GT20 (R = −0.46; *P* < 0.05; supplementary fig. S12, Supplementary Material online).

### Sugar Metabolism Differs Between Species Within *Pisolithus*

**Fig. 5.**
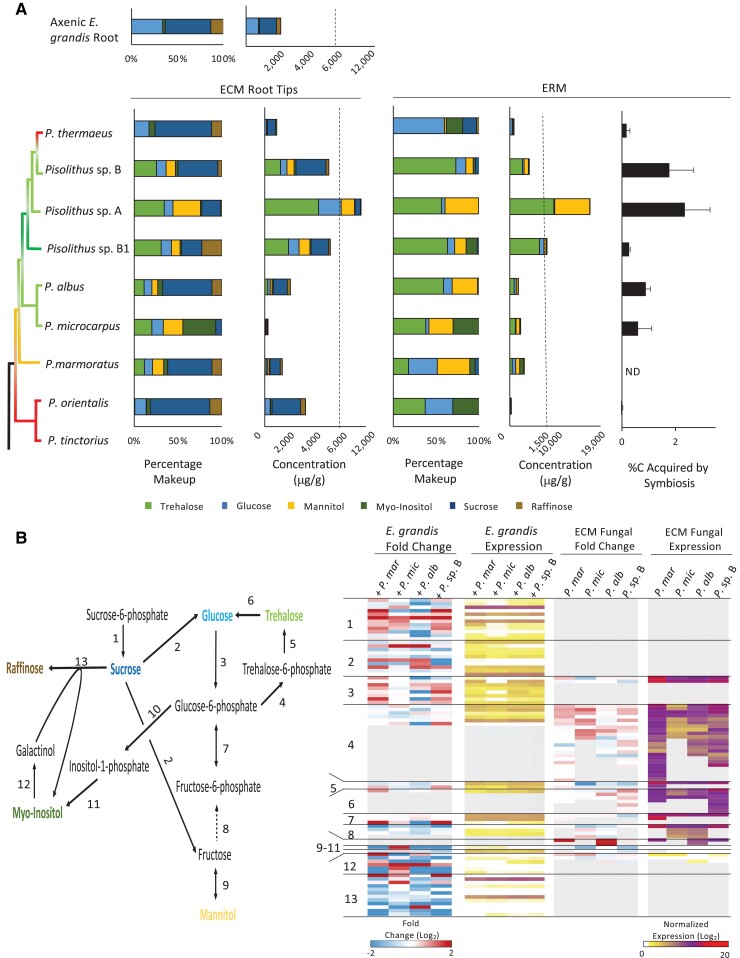
Sugar metabolism during symbiosis between *E. grandis* roots and seven *Pisolithus* species. (*A*) GC-MS quantitation of six key sugars in axenic *E. grandis* roots, *E. grandis* roots colonized by *Pisolithus* (i.e., ECM), or in extra-radical mycelium (ERM). A colored *Pisolithus* phylogenetic tree is utilized to show the relationship between the species, and the colonization potential, defined as the percentage of lateral roots exhibiting Mantle formation, is color coded onto the tree branches whereby <10% colonization = red; 30 < 10% colonized roots is yellow; 50 < 30% colonized roots is light green and >50% colonized roots is dark green. Quantified values are the average of three biological replicates. (*B*) Differential expression of 13 sugar metabolism enzyme families in *E. grandis* or 10 enzymes families in four *Pisolithus* species based on the pictured sugar metabolism tree built from both plant and fungal models. Dashed arrow indicates multiple steps. Each row within the heat map represents the differential gene expression (expression in during colonization vs. axenic growth; blue indicates transcriptomic repression and red represents increased transcription) or normalized expression values in each tissue type (whereby increasing transcript abundance is represented by increasingly dark coloration) for a single gene. Gray boxes indicate no gene found within a given genome. Expression values and gene identity can be found in supplementary table S14, Supplementary Material online.

Due to the differences in CAZymes associated to symbiotic sugar metabolism in *Pisolithus* species versus other fungal genomes considered here, we compared the accumulation of these sugars within symbiotic tissues. When in symbiosis with *E. grandis*, free-sugar profiles of the eight species tested largely reflected their phylogenetic relationship to each other as well as the ability of each *Pisolithus* species to colonize *E. grandis* (fig. 5*a*). For *E. grandis* roots interacting with P. thermaeus and *P. orientalis*, two species which showed fewer than 10% of roots with a visible mantle, sugar profiles in “ECM” tissues closely mirrored those of axenically grown root tips. Conversely, *Pisolithus* sp. A, *Pisolithus* sp. B, and *Pisolithus* sp. B1 exhibited the highest percentage and accumulation of the fungal storage sugar trehalose in ECM tissues. Similar to the results in ECM root tips, extraradical mycelium (ERM) also accumulated higher percentages of trehalose depending upon the level of host colonization (fig. 5*a*). The level of trehalose accumulation in ECM correlated positively to both the percentage of colonized root tips (r(40) = 0.58, *P* < 0.001) and to the percentage of fungal C acquired from plant sugars (r(40) = 0.38, *P* = 0.014) but not to Hartig net depth (fig. 5*a*). It should be noted that the % C acquired reported here is based on pulse labeling, and therefore this latter isolate could have obtained high amounts of host carbon prior to the pulse of labeled CO_2_. Accumulation of sugars such as sucrose and raffinose in the ECM tissues is likely largely driven by the presence of plant cells as host genes encoding the enzymes involved in these biosynthetic pathways are highly induced by symbiosis (fig. 5*b*).

When considering the general trends in sugar accumulation described above, it was noted that *Pisolithus* species with similar abilities to colonize the same host exhibited surprisingly different sugar profiles and accumulation within mycorrhizal root tissues and in ERM. Therefore, we looked deeper into their respective genomes and transcriptomes to understand these differences. At the gene repertoire level, one of the starker differences with relation to sugar metabolism was the finding that *P. microcarpus* had the smallest set of genes encoding α, α-trehalose-phosphate synthase (GT20; 13 genes), whereas other species such as *P. albus* and *Pisolithus* sp. B encoded a greater copy number (19 each; supplementary fig. S9, Supplementary Material online). Similarly, genes encoding α, α-trehalase (GH37) had half the number of these genes in *P. microcarpus* as opposed to *P. albus* (4 vs. 8 gene copies; supplementary fig. S9, Supplementary Material online). However, the genomic expansions of genes within these CAZyme families does not fully explain the differences in sugar accumulation as *P. marmoratus* had an even greater number of GT20 genes (25) whereas having a comparable number of GH37 genes (5; supplementary fig. S9, Supplementary Material online). Therefore, we considered the expression profile of these genes (supplementary file S13, Supplementary Material online). Genes bearing homology to GT20, the step to make the precursor to trehalose (i.e., trehalose synthase 1 [TPS1]; enzyme 4 in fig. 5*b*; supplementary file S14, Supplementary Material online), were highly induced by colonization in *P. microcarpus*. TPS1 genes in *P. albus* and *Pisolithus* sp. B did not show as strong a regulation pattern. Interestingly, an equivalent number of TPS1 genes were induced by colonization in *P. marmoratus* as in *Pisolithus* sp. B despite differing sugar profiles. Of note, the genes showing the closest homology to the following step in this biosynthetic process (i.e., trehalose synthase 2 [TPS2]; enzyme 5 in fig. 5*b*), showed down-regulation or no significant regulation in the *Pisolithus* species that exhibited the greatest accumulation of trehalose. Genes encoding the breakdown of trehalose into glucose (i.e., trehalases; enzyme 6 in fig. 5*b*; supplementary file S14, Supplementary Material online), had greater numbers within the *Pisolithus* sp. B genome as well as a greater number induced by symbiosis when compared with *P. microcarpus*, however, *Pisolithus* sp. B still showed higher levels of trehalose accumulation. Given these conflicting results, we also plotted the normalized expression of these same fungal genes to determine if raw expression of the genes better matched the sugar accumulation quantified (fig. 5*b*; supplementary file S13, Supplementary Material online). Analyzing the data in this manner, we found that there was low expression of TPS1 in *P. microcarpus* and *P. albus*, but higher in *Pisolithus* sp. B, which better matched sugar accumulation. However, *P. marmoratus* also had high expression of these genes. A similar trend was observed in both TPS2 and putative trehalases. Therefore, symbiotic sugar storage in *Pisolithus* cannot be directly correlated to gene copy nor to changes in gene expression but rather to the more complex interplay between these two factors likely combined with post translational modification or with the degree of host colonization.

## Discussion

The evolution of the ECM lifestyle has arisen separately from different saprotrophic lineages many times within the fungal tree of life starting as far back as ∼200 Mya (Tedersoo and Smith 2017; Martin et al. 2017; Strullu-Derrien et al. 2018; Lutzoni et al. 2018; Lebreton et al. 2021). Because of their genetically diverse backgrounds, many comparative genomics studies assess the general shared properties of different fungal lifestyles and have found that there are consistent trends in restrictions and expansions of different gene families related to CAZymes, signaling pathways, and secondary metabolism (Kohler et al. 2015; Murat et al. 2018; Miyauchi, Kiss et al. 2020; Lebreton et al. 2021). Fewer studies, to date, have investigated the degree of genomic and functional diversity within single ECM genera (Hess et al. 2018; Lofgren et al. 2021; Looney et al. 2021; Wu et al. 2022). Our study supports this area of research by studying the globally prevalent, and ecologically important *Pisolithus* genus. We found that, whereas this genus exhibits genomic characteristics previously described as hallmarks of the ECM lifestyle, there was a surprisingly low conservation of coding genes or of transcriptomic regulation when colonizing a common host. We also found evidence that, despite encoding a number of highly expressed SSPs, only a small fraction of these have evolved expression profiles that are responsive to a host root (matching the definition of Mycorrhizal Induced Small Secreted Proteins; MiSSPs; Martin et al. 2008). Further, we found that predictions of functional characteristics such as symbiotic sugar metabolism could not be based purely on genomic analyses, but also relied on transcriptomic, metabolomic, and host compatibility assays. Therefore, intra-genus genomic and functional diversity within ECM fungi may be greater than previously thought, a factor to consider in future when studying the evolution of symbiotic fungi.

Within the study of eukaryotic genomes, the majority of comparative analyses to date are found either within a single species (i.e., pangenomes) or between distantly related genera. In pangenomic studies, a relatively high conservation of orthologous orthogroups has been found with a small number of accessory genes evolved from diverging duplicates of the core genome. This was first studied within yeasts such as *Saccharomyces cerevisiae* (63% of orthologs in the core genome of >1k genomes; Peter et al. 2018), *Cryptococcus neoformans*, and *Candida albicans* (80–90% orthogroups form the core genome; McCarthy and Fitzpatrick 2019). More recent work with multicellular fungi has found a similar level of genomic conservation (*Zymoseptoria tritici*, Badet et al. 2020; *Rhizoctonia solani*, Kaushik et al. 2020; and *Neonectria neomacrospora*, Nielsen et al. 2021). Studies that consider a wider diversity of genomes have also found maintenance of a high percentage of gene orthogroups (Okagaki et al. 2016; Wang et al. 2018). For example, a recent analysis across four genera within the Hypocreales found that a maximum of 7% of orthogroups were unique to a specific genus (Wu and Cox 2021). Compared with these species, our findings of > 60% of the orthogroups being species-specific within *Pisolithus* was surprisingly high.

This difference in gene conservation extended to the transcriptional profile of the five species tested. There was very little overlap in the core orthogroups significantly regulated in each species during interaction with a host. Similar findings were found for *Laccaria* sp. (unpublished) and other mycorrhizal fungi (Mateus et al. 2019). Further, regulation of specific classes of genes may enable fine-tuning of specific fungal-host pairings. Supporting this hypothesis was the differential gene expression of SSPs. Effector-like SSPs, typically <300 amino acids in length and found to be induced during host colonization, have been characterized in mycorrhizal fungi (Kloppholz et al. 2011; Plett et al. 2011, 2014; Pellegrin et al. 2015; Tsuzuki et al. 2016; [Bibr msad045-B105][Bibr msad045-B35]; Plett, Plett et al. 2020) and in pathogenic fungi (Petre et al. 2015; Ahmed et al. 2018; Clark et al. 2018; Irieda et al. 2019; Madina et al. 2020) and shown to be essential for overcoming host defenses to achieve colonization. Our analyses here would demonstrate that of 129 *Pisolithus* accessory and core SSP orthogroups, only 6–20% were significantly induced by the presence of a host (i.e., conforming to the definition of a putative effector-like MiSSP in mycorrhizal fungi), whereas the remaining majority of SSPs were constitutively expressed regardless of growth condition. Therefore, we must consider both the genomic landscape of mycorrhizal fungi as well as their transcriptomic regulations when colonizing a host plant to better understand the number and diversity of pathways that have evolved to support beneficial plant–fungal interactions.

When we consider specific annotations of the orthogroups found within the core versus specific genome, we found that they were characterized by different CAZymes. Similarly, McCarthy and Fitzpatrick (2019) found that carbohydrate metabolism genes across four genera of yeast were more likely to be found within the accessory genome suggesting a specific evolutionary pressure on this pathway within fungi. The genomic repertoire of CAZymes was recently proposed to be reflective of the age of the symbiosis along the saprotrophy to symbiosis continuum, whereby ECM fungi have more of these enzymes than AM fungal genomes but fewer than orchid mycorrhizae (Miyauchi, Kiss et al. 2020). However, our results would indicate that even between ECM fungi there can be large differences in the classes of CAZymes that have been retained or have diverged. Specifically, *Pisolithus* is different from other ECM fungi sequenced to date with respect to enzymes within the trehalose pathway. As the main storage form of symbiotically acquired sugar (Martin et al. 1998; Lopez et al. 2007), trehalose biosynthesis and metabolism is core to the mutualistic aspect of the ECM lifestyle. Thus, our results would suggest that within *Pisolithus*, differential genomic profiles in specific gene classes/functions form an important foundation to speciation and divergence in host specificity.

Upon receipt of glucose from their plant partners, ECM fungi typically synthesize this substrate into trehalose thereby maintaining a strong carbon sink towards the mycorrhizal root tip (Martin et al. 1998; Lopez et al. 2007). This metabolic process has been found to occur preferentially within the hyphae of the Hartig net after which trehalose is exported to the ERM (Lopez et al. 2007). Our finding that the profile of trehalose-related metabolic enzymes in the *Pisolithus* genomes set them apart from other ECM fungal genomes sequenced to date, and that there were large differences in the numbers of genes associated with trehalose biosynthesis between *Pisolithus* species, led us to question if the genomic and/or transcriptomic profiles of these species predicted their sugar metabolism. Interestingly, there appeared to be no correlation between genomic potential for trehalose biosynthesis (i.e., gene copy number) and the quantified trehalose metabolite. This is similar to previous work that we have done in *P. microcarpus* where we found no relationship between the transcriptional profile of sugar biosynthetic enzymes and trehalose concentration (Plett et al. 2021). Our findings would suggest that the symbiotic compatibility between *Pisolithus* species (or even intra-species variation based on genotypic variation) and a given host as defined by Hartig net depth or the transfer of plant C to the fungus, is a better predictor of sugar metabolism and accumulation in the ERM. These findings have implications in our understanding of mechanisms governing carbon cycling and sequestration in forest soils due to ECM fungi. Although typically ECM fungi are thought to increase carbon sequestration (Agerer 2001; Clemmensen et al. 2013; Averill et al. 2014; Averill and Hawkes 2016), the long-term stability of sugars they produce may also contribute to successful storage of carbon belowground. The discovery of mycorrhizal-associated sugars such as trehalose remaining stable in Cretaceous and Miocene deposits (Marynowski et al. 2019) would support this hypothesis. Therefore, in addition to considering the species of fungi and their degree of mycelial production in forest soils, we must also understand that markers for carbon sequestration in forest soils include the degree of symbiotic compatibility between the ECM fungi and plant hosts present.

Overall, the analysis of these nine *Pisolithus* genomes highlights the unexpected level of variation seen between closely related species, in terms of genome size and content, transcriptional regulation and metabolic potential. An appreciation of what is “core” to the ECM lifestyle will rely on increased numbers of sequenced genomes and characterization of fungi beyond model species to encompass the true functional potential of this evolutionarily diverse group of fungi. Further, as *Pisolithus* is widely distributed and shows a high degree of diversity between species, it will be of interest in future to determine if there is a correlation between the degree of intra-genus genomic and functional variation seen within an ECM lineage and the extent of its global distribution.

## Materials and Methods

### Fungal Isolation, Growth, and DNA Extraction for Sequencing

Dikaryotic pure cultures of *Pisolithus* were used in this study. Fungal cultures were obtained from Australia (*Pisolithus* sp. B1, *P. albus* SI12, *P. microcarpus* MW3; Scientific License S13146; Hawkesbury Institute for the Environment Fungal Culture Collection), New Zealand (*P. thermaeus*, *Pisolithus* sp. A, *Pisolithus* sp. B, *P. marmoratus* 16; Landcare Research National Authorization Number 38493-FLO), Brazil (*P. microcarpus* 441), USA (*P. tinctorius* s.str. Marx 270) or Japan (*P. orientalis* OTSU; NITE Biological Resource Centre). In preparation for genomic DNA extraction, all isolates were inoculated into sterile liquid MMN (3.78 mM (NH_4_)_2_PO_4_; 2.2 mM H_2_KPO_4_; 0.568 mM MgSO_4_ 7H_2_O; 55.5 mM C_6_H_12_O_6_; 0.45 mM CaCl_2_; 0.428 mM NaCl; 0.0167 mM ZnSO_4_; 0.004 mM thiamine; 0.065 mM citric acid, 0.0362 mM Fe-EDTA in a 1.3% agar solution at a pH 5.5) and grown in a static incubator at 25 °C for three weeks. Flash frozen mycelium was then ground in liquid nitrogen and extracted as per Kohler et al. (2015). To verify the fungal species, the ITS sequence was amplified using MyTaq (Bioline) as per manufacturer's instructions using the fungal primers ITS1F and ITS4 (Gardes and Bruns 1993; Anderson et al. 2001; supplementary figure S13, Supplementary Material online). PCR amplicons were purified using a Wizard SV PCR and Gel cleanup kit (Promega) and sequenced at the Hawkesbury Institute for the Environment sequencing facilities (Sydney, Australia). In preparation for genomic DNA extraction, all isolates were inoculated into sterile liquid MMN (3.78 mM (NH_4_)_2_PO_4_; 2.2 mM H_2_KPO_4_; 0.568 mM MgSO_4_ 7H_2_O; 55.5 mM C_6_H_12_O_6_; 0.45 mM CaCl_2_; 0.428 mM NaCl; 0.0167 mM ZnSO_4_; 0.004 mM thiamine; 0.065 mM citric acid, 0.0362 mM Fe-EDTA in a 1.3% agar solution at a pH 5.5) and grown in a static incubator at 25 °C for three weeks. Flash frozen mycelium was then ground in liquid nitrogen and extracted as per Kohler et al. (2015).

### Confirmation of Identity (ITS-based Phylogeny)

Sequences representing a broad range of species within *Pisolithus* were retrieved from GenBank and UNITE following Lebel et al. (2018), for comparison to those generated in this study. The dataset comprised 158 ITS sequences, consisting of 599 bp, with *P. abditus* included as outgroup. Phylogenetic analysis was performed with Maximum Likelihood (ML) in RAxML v. 8.2.12 (Stamatakis 2014) using standard algorithm and 1,000 bootstrap replicates on the CIPRES Science Gateway v. 3.3 (Miller et al. 2010). The resulting tree (supplementary fig. S13, Supplementary Material online) was visualized using FigTree v1.3.1 (Rambaut 2009).

### Genome Sequencing and Assembly

The nine *Pisolithus* genomes reported in this study were sequenced with the Pacific Biosciences platform (“PacBio”), assembled with FALCON (Chin et al. 2016), and annotated with the JGI Annotation Pipeline (Grigoriev et al. 2014; Kuo et al. 2014) aided by the nine matching transcriptomes. For genome sequencing, 1–25 µg of gDNA was used to generate each library. The DNA was sheared to 10 kb or 30 kb fragments using Covaris g-TUBE or Megaruptor. The sheared DNA was treated with exonuclease to remove single-stranded ends and DNA damage repair mix followed by end-repair and ligation of blunt adapters using SMRTbell Template Prep or Express Kit 1.0 (Pacific Biosciences). The final library was size selected with BluePippin (Sage Science) at 6 kb, 10 kb, or 20 kb cutoff size and purified with AMPure PB beads. PacBio Sequencing primer was then annealed to the SMRTbell template library and sequencing polymerase was bound to them using Sequel Binding kit 2.0 or 3.0. The prepared SMRTbell template libraries were then sequenced on a Pacific Biosystem's Sequel sequencer using v3 sequencing primer, 1 M v2 SMRT cells, and Version 2.1 or 3.0 sequencing chemistry with 1 × 360 & 1 × 600 sequencing movie run times. Sequenced transcriptomes were used to assess the completeness of genome assemblies and to seed and assess genome annotations. All transcriptomes were sequenced using Illumina RNA-Seq with polyA selection, whereas the *P. microcarpus* and *P. tinctorius* s.str. transcriptomes were additionally sequenced with PacBio. All Illumina reads were assembled into RNA contigs using Trinity (Grabherr et al. 2011) and all PacBio reads were clustered using Iso-Seq (Gordon et al. 2015). Stranded cDNA libraries were generated using the Illumina Truseq Stranded mRNA Library Prep kit as per manufacturer's instructions. The prepared libraries were quantified using KAPA Biosystem's next-generation sequencing library qPCR kit and run on a Roche LightCycler 480 real-time PCR instrument. Sequencing of the flow cell was performed on the Illumina HiSeq2500 sequencer using HiSeq TruSeq SBS sequencing kits, v4, following a 2 × 150 indexed run recipe. For re-annotation of improved genomes of *P. microcarpus* and *P. tinctorius* s.str., their transcriptomes were also sequenced using PacBio Iso-Seq. For this, full-length cDNA was synthesized using template-switching technology with SMARTer PCR cDNA Synthesis kit (Clontech). The prepared SMRTbell template libraries were then sequenced on a Pacific Biosystem's Sequel sequencer using v3 sequencing primer, 1 M v3 SMRT cells, and Version 3.0 sequencing chemistry with 1 × 1,440 sequencing movie run times. We performed a stringent quality control to avoid any spurious contaminating sequences in the genome assemblies. Any mitochondrial or non-target contaminant contigs were filtered from the final assembly before gene annotation. Contaminants were identified using the following post-assembly methods: BLAST against NCBI databases (nt, refseq.fungi, ref_prok_rep, mitochondrial, NCBI gcontam, and Pacbio 2/4K-control sequences), principal component analysis of tetramer nucleotide frequency, contig length × GC × aligned read coverage, aligned the assembled rDNA IT sequences using BLAST against the UNITE database (https://unite.ut.ee). Each genome was assembled with FALCON (Chin et al. 2016) and annotated using the JGI Annotation Pipeline (Grigoriev et al. 2014; Kuo et al. 2014), which detects and masks repeats and TEs, predicts genes based on transcriptome or nr protein evidence, characterizes each conceptually translated protein with sub-elements such as domains and signal peptides, chooses a best gene model at each locus to provide a filtered working set, orthogroups the filtered sets into draft gene families, ascribes functional descriptions such as GO terms and EC numbers, and creates a JGI genome portal with tools for public access and community-driven curation of the annotation (Grigoriev et al. 2014; Kuo et al. 2014). The *P. microcarpus* and *P. tinctorius* s.str. annotation inputs were further augmented with the Gene Catalog gene models and transcriptomes of the v1 genome projects (Kohler et al. 2015).

### Whole Genome Phylogenetic Tree Construction

JGI unmasked genome assemblies and protein sequences were downloaded from JGI MycoCosm website (https://mycocosm.jgi.doe.gov). Orthologous genes among the selected fungi were identified using FastOrtho with the parameters set to 50% identity, 50% coverage, inflation 3.0 (Wattam et al. 2014). Orthogroups with single-copy genes were identified and aligned with MAFFT 7.221 (Katoh and Standley 2013), ambiguous regions (containing gaps and poorly aligned) were eliminated, and 642 single-gene alignments were concatenated with Gblocks 0.91b. A phylogenetic tree was constructed with RaxML 7.7.2 (Stamatakis 2014) the standard algorithm, the PROTGAMMAWAG model of sequence evolution and 1,000 bootstrap replicates.

### Comparative Genomic Features

Functional annotation sets were integrated using the following databases: carbohydrate active enzyme (CAZy, Lombard et al. 2014), GO (The Gene Ontology Consortium 2015), Kyoto Encyclopedia of Genes and Genomes (KEGG, Ogata et al. 1999), EuKaryotic Orthologous Groups (KOG; Tatusov et al. 2003), Protein families (PFAM; Finn et al. 2016). KOG, GO, KEGG, PFAM were obtained from MycoCosm, JGI. The latest CAZyme annotation was obtained from the CAZy team (http://www.cazy.org/). Statistics of JGI genome assemblies (i.e., N50, number of genes and scaffolds, genome size) were obtained from JGI MycoCosm (https://mycocosm.jgi.doe.gov). Genome completeness with single-copy orthologs was calculated using BUSCO v3.0.2 with default parameters (basidiomycota_odb9 database; Simão et al. 2015). The coverage of TEs in genomes was calculated and visualized using the visual omics pipeline Transposon Identification Nominative Genome Overview (TINGO; Morin et al. 2019). Secreted CAZymes, lipases, proteases, and SSPs (< 300 amino acids) were identified (Pellegrin et al. 2015). We calculated, visualized, and compared the count and ratio of total (present in the genomes) and predicted secreted CAZymes, lipases, proteases, and SSPs as a subcategory. We calculated the total count of the followings using both all and theoretically secreted plant cell wall degrading enzymes. Global trends of ecological groups were evaluated using Non-metric Multi-Dimensional Scaling (NMDS) with the count of total and predicted secreted CAZymes. The dissimilarities among the ecological groups were calculated and the relationship was converted into distances in 2D space with the function metaMDS in R package Vegan (Oksanen et al. 2019). We examined the total and predicted secreted counts of CAZymes, lipases, proteases and SSPs. Statistically significant ecological groups were determined with pairwise PERMANOVA. The percentage of variances (R2) was estimated for selected genomic features explained by variables including ecological groups, phylogenetic distances and the genome coverage of Tes with permutational multivariate analysis of variance (PERMANOVA). Detailed procedures are as previously described (Miyauchi, Kiss et al. 2020). We examined associations of genomic features with the R packages PCATOOLS (Blighe and Lun 2020). Output files generated above were combined and visualized with Proteomic Information Navigated Genomic Outlook (PRINGO; Miyauchi, Kiss et al. 2020). We statistically tested the genomic features of *Pisolithus* species compared with other fungi. We conducted ANOVA with Tukey's honest significance test and Kruskal–Wallis test for the TE coverage in the genomes and the count of genes coding for GT2, GT4, GT20, GT90, respectively, with R package agricolae (Mendiburu and Yaseen 2020). The proteomes of the nine *Pisolithus* species were clustered using FASTORTHO (Wattam et al. 2014) with the following parameters: 50% identity and 50% coverage. The core genome consists of genes found in all nine sequenced genomes of *Pisolithus* species, whereas accessory genes are found in at least two species. Finally, species-specific genes are found in only one species. The definition of “core”, “accessory”, and “specific” genes are used based on this 9 genome comparison throughout the study. Phylogenetic trees with protein sequences of SSPs were constructed with five *Pisolithus* species that were used in transcriptomic sequencing. Protein sequences, which were obtained from JGI protein assemblies, were aligned with MAFFT 7.475 (Katoh and Standley 2013), and trees were built with Fast Tree 2.1.10 (Price et al. 2009). We calculated differential gene expression values with DESeq2 (Love et al. 2014).

### TE-Gene Distance Analysis

We measured the mean TE-gene distances with statistical support by comparing the locations of observed genes and Tes and 10,000 null hypothesis genome models made by randomly reshuffling the locations of genes. The probability (*P* value) of mean TE-gene distances was calculated with R package, regioneR (Gel et al. 2016). Across the entire genomic space, the proximity of coding genes to repeat elements was calculated (supplementary fig. S4, Supplementary Material online). The distance of genes to nearest repeat elements was defined as the length from the start of genes to; i) the start of nearest repeats in the downstream; ii) the end of nearest repeats in the upstream. We used such tactics to estimate repeat elements located outside genes as well as ones overlapped to genes. The distance of genes to nearest repeat elements within 4.5 kb was defined as “close proximity” because coding genes tend to be smaller than 4 kb (fig. 3*a*). The process above was conducted with the visual pipeline Synteny Governed Overview (SynGO; Looney et al. 2021). We performed ordination analyses and estimated Pearson correlation to examine associations between TE-gene distance of various genes coding for different protein types with R package PCAtools and Ggally (Blighe and Lun 2020; Schloerke et al. 2021).

### Host Colonization Microcosms

To understand how the different fungal isolates colonized a common host, all species used here (excluding *P. tinctorius* s.str. as it typically colonizes gymnosperm, Fagaceae or Cistaceae hosts) were put into contact with *Eucalyptus grandis* in a microcosm as described previously (Plett et al. 2019; unless otherwise noted). The medium used was reduced nutrient MMN (1.9 mM (NH_4_)_2_PO_4_; 1.1 mM H_2_KPO_4_; 0.28 mM MgSO_4_ 7H_2_O; 5.5 mM C_6_H_12_O_6_; 0.23 mM CaCl_2_; 0.22 mM NaCl; 0.008 mM ZnSO_4_; 0.002 mM thiamine; 0.033 mM citric acid, 0.018 mM Fe-EDTA in a 1.3% agar solution at a pH 5.5). Isolates used were *P. thermaeus* 76C, *P. marmoratus* 721, *P. microcarpus* 441 (for transcriptomic analysis in figs. 2 and 3 colonizing *E. globulus*) or *P. microcarpus* MW3 (comparable genetically to isolate 441 for figs. 2*a* and 5, colonizing *E. grandis*), *Pisolithus* sp. A, *Pisolithus* sp. B, *Pisolithus* sp. B1, *P. albus* SI12, and *P. orientalis* OTSU. Differences in isolates used for genome sequencing and other experimental work was due to the fact that certain isolates of *Pisolithus* are short-lived in culture and the original isolates were dead. Replacement isolates were chosen based on ITS phylogeny as being most closely related to the original sequenced isolate (supplementary fig. S13, Supplementary Material online). Plates containing the fungal isolates alone without a seedling were similarly prepared for FLM controls. Five and three days prior to harvest, the seedlings were pulse labeled with ^13^CO_2_ according to the methods of Plett, Singan et al. (2020). Root systems were scored for % mantle formation (number of roots exhibiting a fungal mantle, i.e., a short, thickened appearance with rounded tip divided by the total number of lateral roots in contact with fungi) at the end of the experiment (14 days of co-culture). Root tips from each plant were excised and fixed in 4% paraformaldehyde at 4 °C for Hartig net analysis as reported in Plett and colleagues (2021). Colonized roots were also excised from each plant and divided into two samples: one sample for RNA extraction and transcriptomic sequencing and one sample for sugar analysis. All leaves from each seedling were collected for 13C isotopic analysis as a photosynthesis control. Extraradical mycelium (ERM) was also sampled for sugar and isotopic analysis. Finally, FLM from the fungal-only control plates was sampled for RNA extraction, sugar, and isotopic analysis. Both samples for RNA and for sugar analysis were flash frozen in liquid nitrogen and stored at −80 °C until extraction, whereas tissues for isotopic analysis were dried overnight at 40 °C.

### RNA Extraction, Sequencing, Alignment, and Differential Gene Analysis

RNA from colonized root tip and FLM samples was extracted using the ISOLATE II Plant miRNA kit (Bioline) as per the manufacturer's instructions. RNA for three to four biological replicates of each condition was sequenced at JGI according to the methods of Plett, Singan et al. (2020). Quality trimmed raw reads were aligned to the respective *Pisolithus* reference transcripts available at JGI (https://mycocosm.jgi.doe.gov) using CLC Genomics Workbench v20 (Qiagen). The following CLC genomic workbench parameters were used for read mapping: minimum length fraction 0.9, minimum similarity fraction 0.8, mismatch cost = 2, insertion cost = 3, deletion cost = 3, and the maximum number of hits for a read was set to 10 (supplementary table S1, Supplementary Material online). The unique and total mapped reads number for each transcript were determined and then we normalized read counts and calculated differential expression of the genes among the conditions with the DESeq2 package in R (Love et al. 2014). Genes with statistically significant differences in expression (differentially expressed genes; DEGs) were selected based on FDR adjusted *P* value < 0.05. All procedures were performed with SHIN + GO (Miyauchi et al. 2016, 2017, 2018; Miyauchi, Hage et al. 2020).

### Sugar Quantification and C Acquisition Calculation

Sugar quantification was performed as previously outlined in Plett and colleagues (2021) whereby between 10 and 50 mg (FW) of colonized roots or extraradical mycelium (ERM) were ground and extracted with 250 μl methanol followed by 250 μl of water at room temperature. The extractions were mixed, dried, and derivatized with N, O-Bistrifluoroacetamide + 1% trimethylchlorosilane (BSTFA + TMCS, for GC derivatization, Supelco; Mogosanu et al. 2011). Samples were analyzed using Agilent Technologies 5975C Gas Chromatograph-Mass Spectrometer System operated in Select Ion Monitoring (SIM) mode. The GC was equipped with an Agilent J&W HP-5 ms capillary column; 30 m × 0.25 mm × 0.25 µm with the following temperature program: 70 °C for 1 min, followed by increases of 7 °C/min to 320 °C, held for 1 min. One µL of sample was injected into the GC inlet (260 °C) in split mode (20:1). The temperatures of the MS ion source and quadrupole were set at 230 °C and 150 °C, respectively. Sugars were identified based on pure compound standards (Sigma-Aldrich). A quantitative analytical method in SIM mode was used to determine sugars concentration in the samples, responses of the most abundant ions for all compounds were quantitated.

To assess the percentage of fungal C acquired by symbiosis during pulse labeling, the leaf and ERM tissue collected and dried was sent for 13C stable isotope analysis by IRMS (University of Queensland, Stable Isotope Geochemistry Laboratory). Calculations of % C acquired by symbiosis were done according to the methods of Plett, Singan et al. (2020) and were based on the difference in δ13C values between ERM and FLM tissues, relative to the δ13C level of the corresponding host plant leaves (*n* = 6). The calculated % C acquired here does not reflect total C transferred to the fungus across the whole of the experiment, but rather is indicative of transfer during a specific time period of symbiosis.

## Supplementary Material

msad045_Supplementary_Data
