# Supplementary material for: Speciation Underpinned by Unexpected Molecular Diversity in the Mycorrhizal Fungal Genus Pisolithus
Source: Mol Biol Evol. 2023 Feb 22;40(3):msad045. doi: 10.1093/molbev/msad045 (PMC10066745; doi:10.1093/molbev/msad045)
Supplement: msad045_Supplementary_Data [file msad045_supplementary_data.zip › Supplemental_Figures.pdf]

Supplemental Figures

Speciation underpinned by unexpected molecular diversity in the mycorrhizal fungal genus Pisolithus

Jonathan M Plett*†, Shingo Miyauchi*, Emmanuelle Morin, Krista Plett, Johanna Wong-Bajracharya, Maira de Freitas Pereira, Alan Kuo, Bernard Henrissat, Elodie Drula, Dominika Wojtalewicz, Robert Riley, Jasmyn Pangilinan, William Andreopoulos, Kurt LaButti, Chris Daum, Yuko Yoshinaga, Laure Fauchery, Vivian Ng, Anna Lipzen, Kerrie Barry, Vasanth Singan, Jie Guo, Teresa Lebel, Mauricio Dutra Costa, Igor V. Grigoriev, Francis Martin*, Ian C Anderson*, Annegret Kohler

* Denotes equal author contribution

†Corresponding Author: Jonathan M Plett, j.plett@westernsydney.edu.au

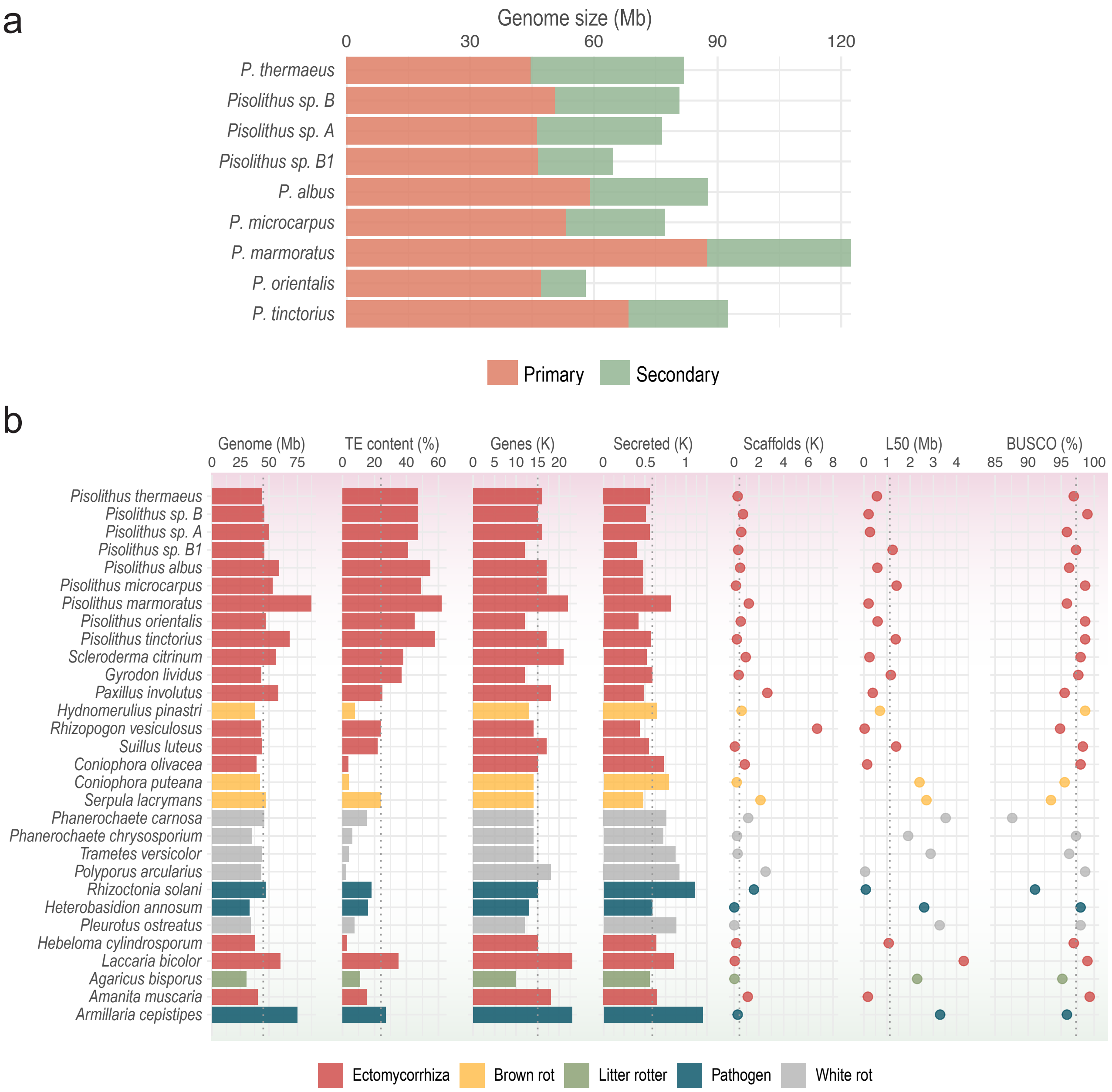

Figure S1. The size of primary and secondary scaffolds in nine *Pisolithus* species. (a) Primary and secondary scaffolds of genome assemblies are in color. See Table S1. (b) The species are in the evolutionary order. Fungal ecology is in color. Median values are in dotted line. Genome: Genome size. TE content: The coverage of transposable elements in the genomes. Genes: The number of genes. Secreted: The number of theoretically secreted proteins (see Methods). Scaffolds: The number of scaffolds. L50: N50 length. BUSCO: Genome completeness. See Table S2.

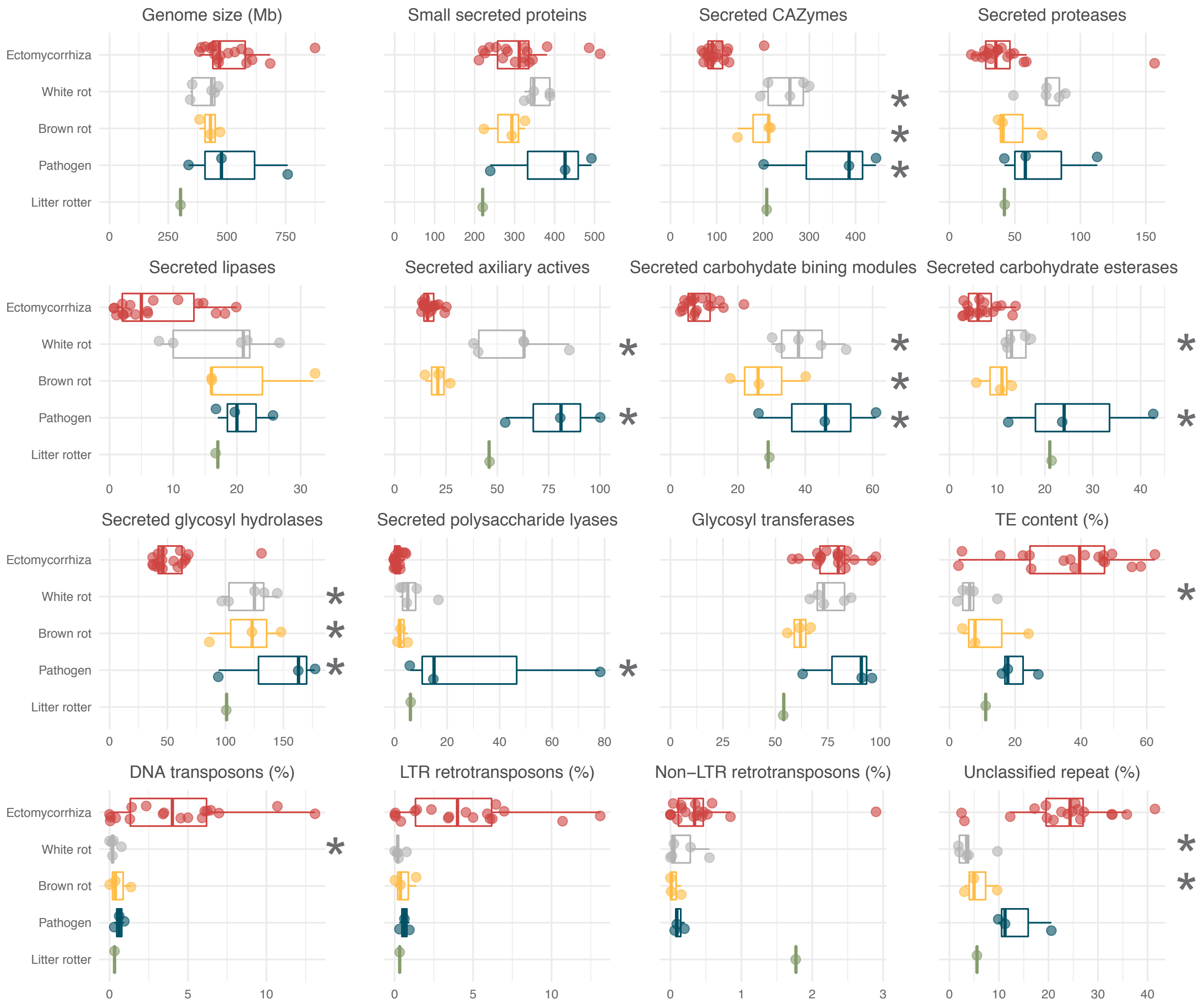

Figure S2. Distribution of genomic features per ecological group with 30 species. Asterisks indicate significantly different ecological groups compared with ectomycorrhizal (ECM) symbionts (p-value < 0.05; Pair-wise PERMANOVA). See Table S3.

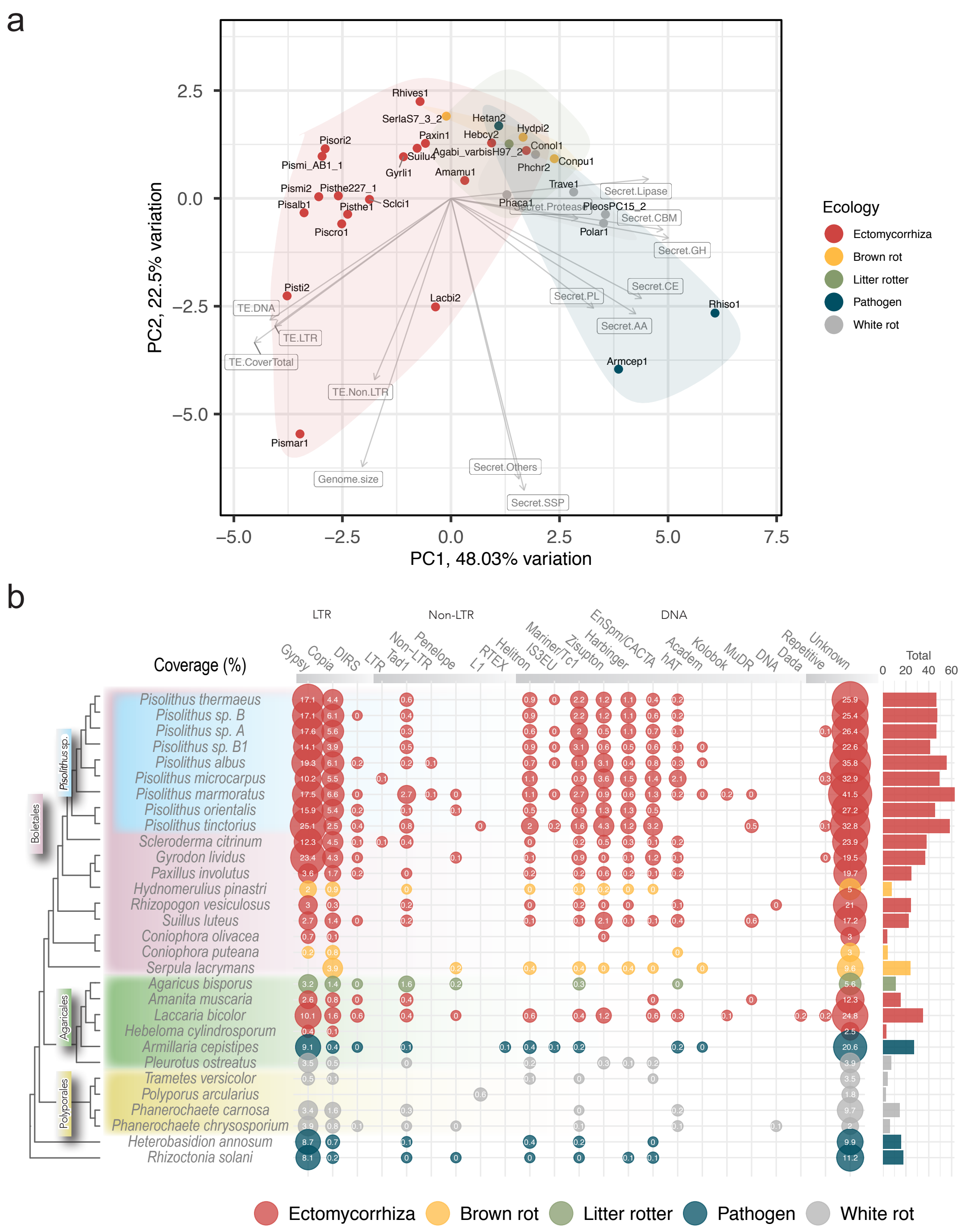

Figure S3. Overview of genomic features and statistics. (a) Two dimensional map with genomic features of 30 fungi with PC1 and 2. Principal components were calculated with the selection of genomic features (i.e. the size of genomes, the number of genes for various types of secreted proteins, the genome coverage of different transposable element families). The lifestyle of fungi are in color. Arrows show highest loadings of variables driving variation in principal components. (b) Coverage of transposable elements identified. LTR: Long terminal repeat retrotransposons. Non-LTR: Non-long terminal repeat retrotransposons. DNA: DNA transposons. Repetitive: Simple repeats. Unknown: Unclassified repeated sequences. The bubble size is proportional to the coverage of each of transposable elements (showing inside the bubbles). The right bars show the total coverage per genome.

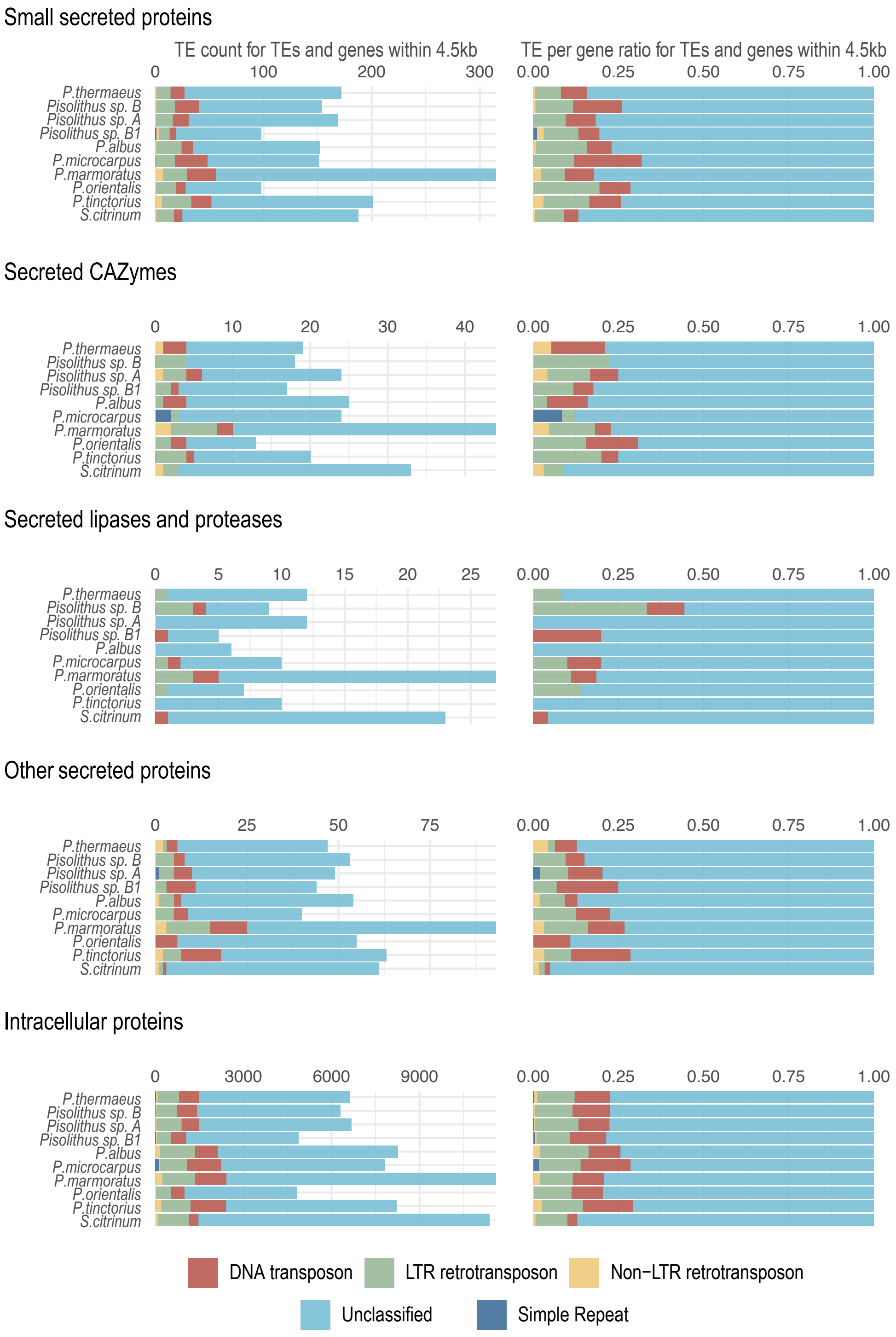

Figure S4: Genes in close proximity to repeat elements for ten species. The count of repeat elements near genes within 4.5 kb. The distance threshold of 4.5 kb was determined based on the observation that most genes are less than 4 kb long (Fig 3a) and 0.5 kb for additional distance to repeat elements.

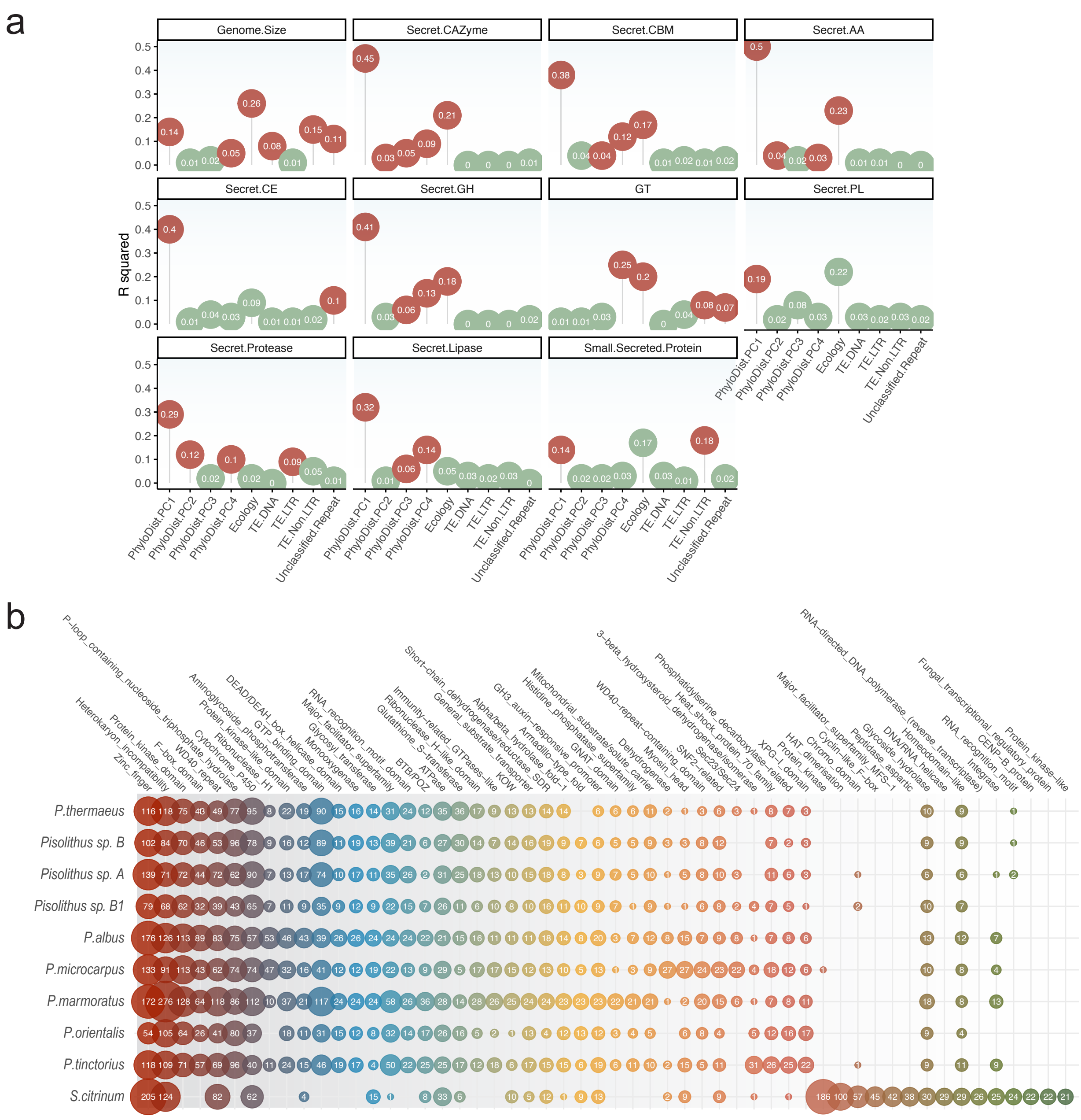

Figure S5. Transposable elements associated with differences in coding space within *Pisolithus* genomes. (a) Proportion of variation explained in selected genomic features with 30 fungi. Circles with numbers inside indicating R squared values for variables tested. Circles in red indicate significant variables (p-value < 0.05; PERMANOVA model, Genomic features ~ Phylogeny + Ecology + TE types). PhlyloDist.PC1 to 4: Major principal components covering over 80% of variation in phylogenetic distances. Ecology: Fungal lifestyle. DNA/LTR/Non-LTR: TE family coverage in the genomes. See Table S11. (b) Highly frequent PFAM terms for genes near repeat elements. PFAM terms (1 term per gene) were extracted and counted. Highly frequent PFAM terms from the genes near TEs/repeats showed some interesting terms such as GH3 auxin responsive promoter and glycosyl transferases, which may be relevant to ECM development.

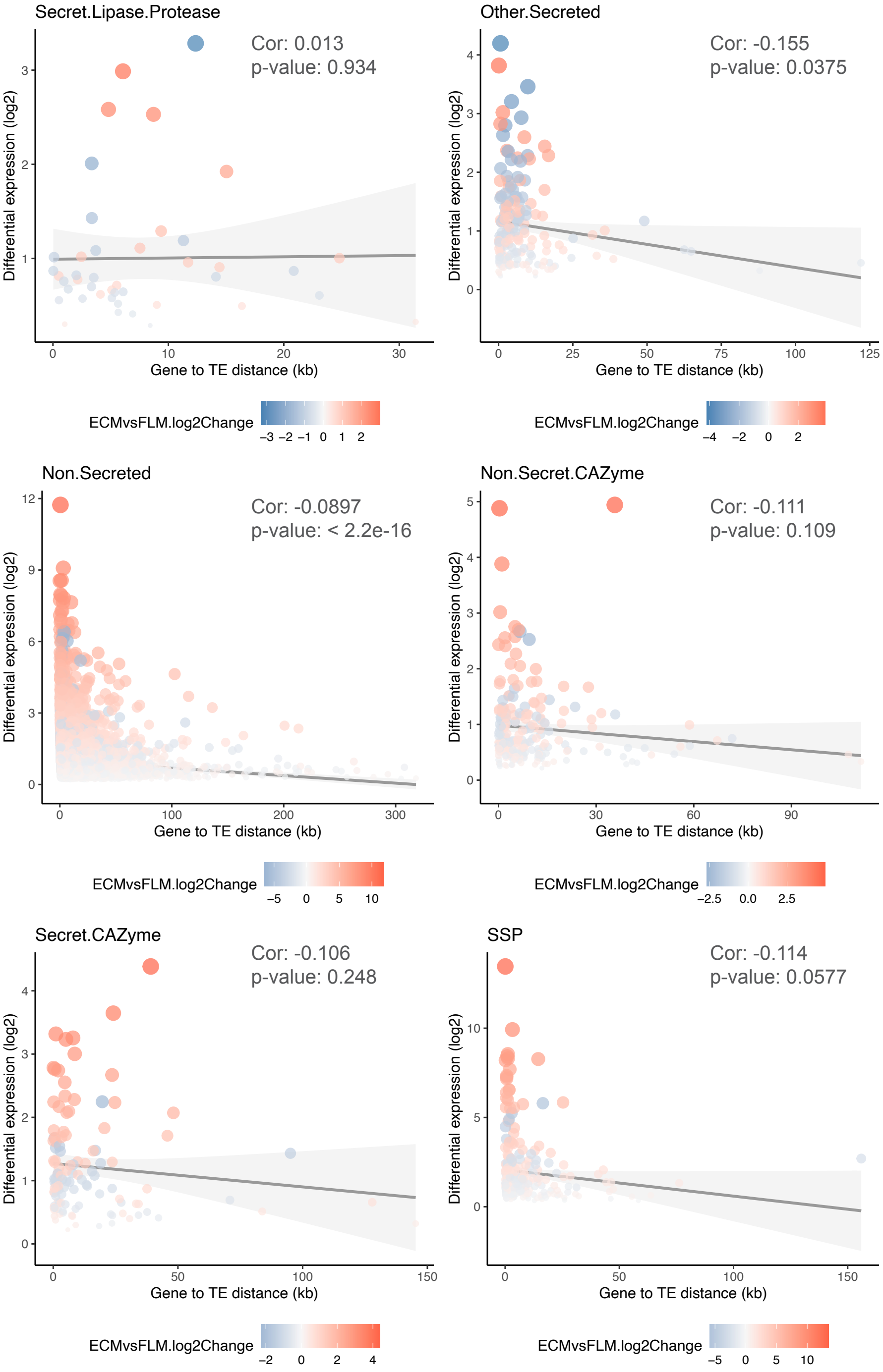

Figure S6: Correlations of the distance of genes to transposable elements and differential expression of genes for various protein types. Gradient colors with the size of circles correspond to differential gene expression values (in log2) for the ectomycorrhizal formation compared to free living mycelia. The following five *Pisolithus* species were used *P. tinctorius, P. marmoratus, P. microcarpus, P. albus, Pisolithus sp. B*. The numbers in the top right corner indicate Pearson correlation coefficient. Statistically significant protein categories are indicated with p values. Regression lines with grey area show estimated liner models with 95% confidence interval.

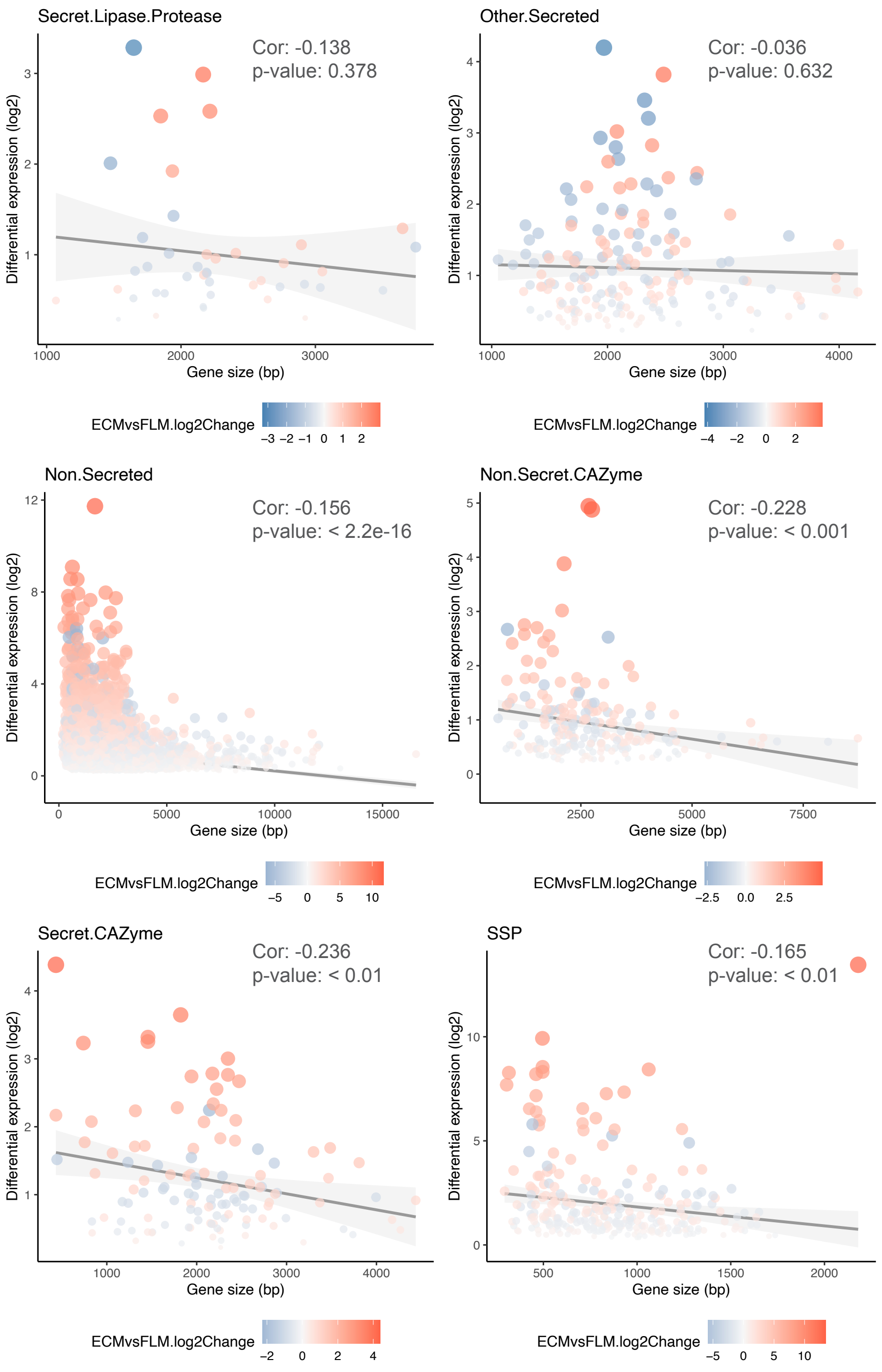

Figure S7: Correlations of gene size and differential expression of genes for various protein types. Gradient colors with the size of circles correspond to differential gene expression values (in log2) for the ectomycorrhizal formation compared to free living mycelia. The following five *Pisolithus* species were used *P. tinctorius, P. marmoratus, P. microcarpus, P. albus, Pisolithus sp. B*. The numbers in the top right corner indicate Pearson correlation coefficient. Statistically significant protein categories are indicated with p values. Regression lines with grey area show estimated liner models with 95% confidence interval.

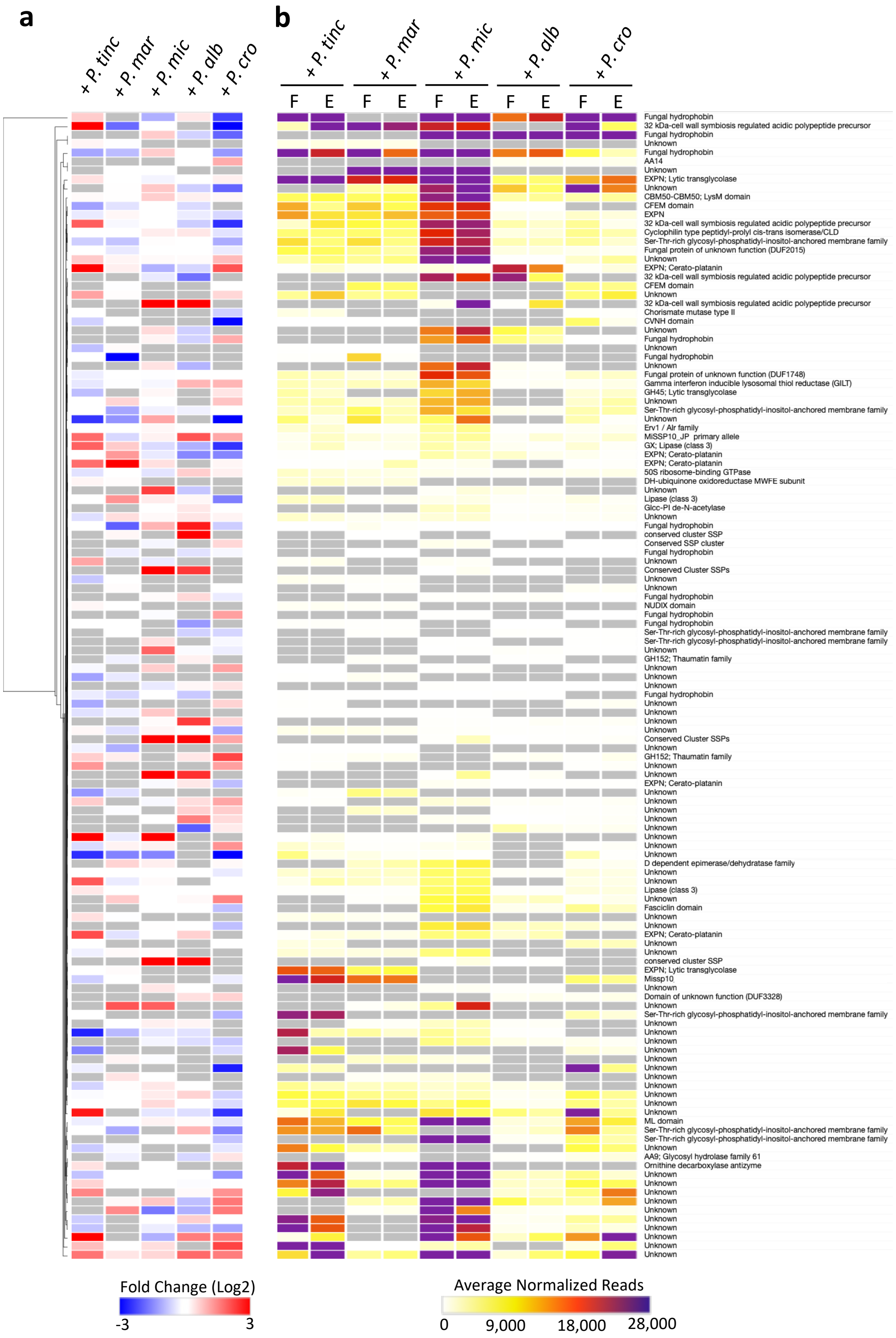

Figure S8: Expression of core and accessory *Pisolithus* Small Secreted Proteins (SSPs). a. Differential expression (log2) of the ectomycorrhizal condition compared to the control. b. Normalized transcription of ectomycorrhizal (E) and free living mycelium (F) conditions. Functional annotation terms are shown on the Y-axis. P. tinc: *Pisolithus tinctorius.* P. mar: *Pisolithus marmoratus*. P. mic: *Pisolithus microbarpus*. P. alb: *Psolithus albus.* P.cro: *Pisolithus sp. B.*

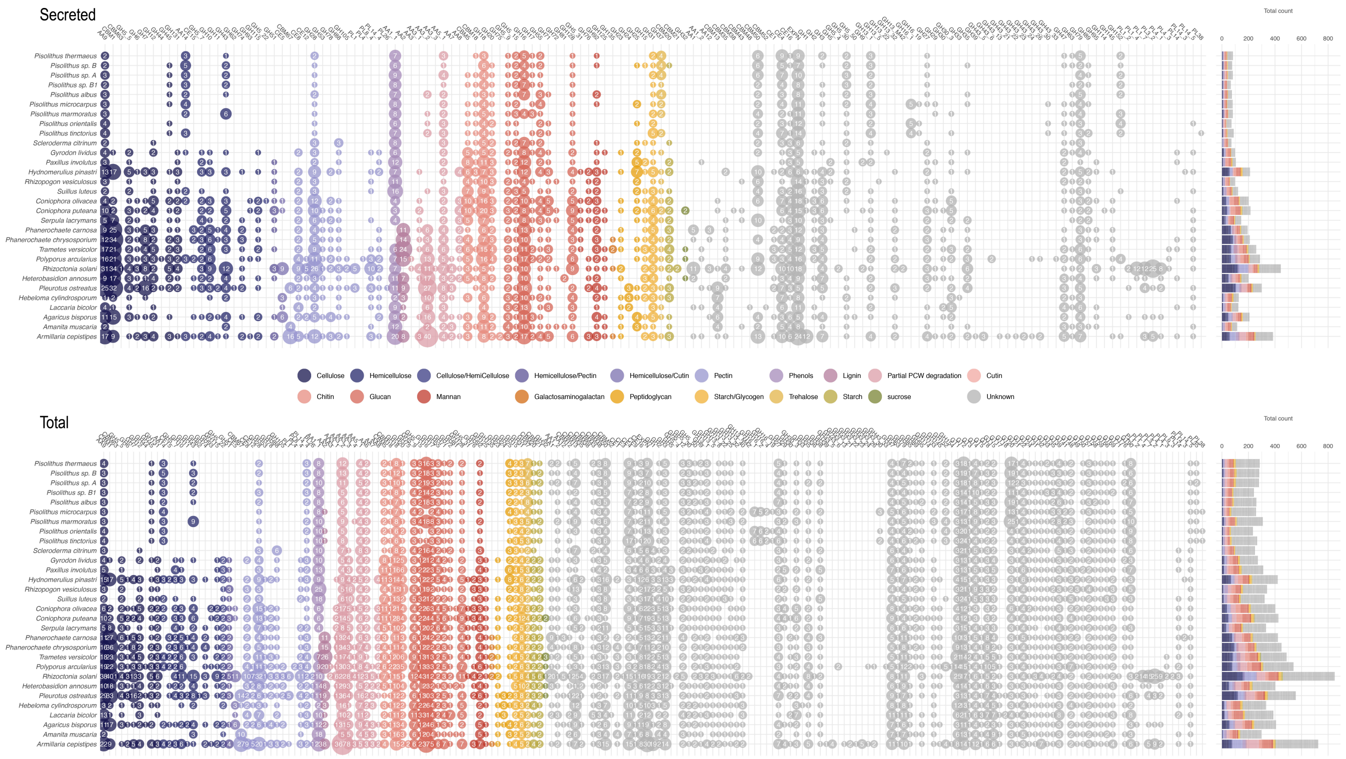

Figure S9: Overview of presence/absence of CAZyme domains. The count of CAZyme domains theoretically secreted and total (extracellular and intracellular combined) in the genomes. The bars on the right show the count of CAZyme domains in total. 30 fungal species are in the evolutionary order. Substrate specificities are in colors. See Table S12 for the count of CAZymes.

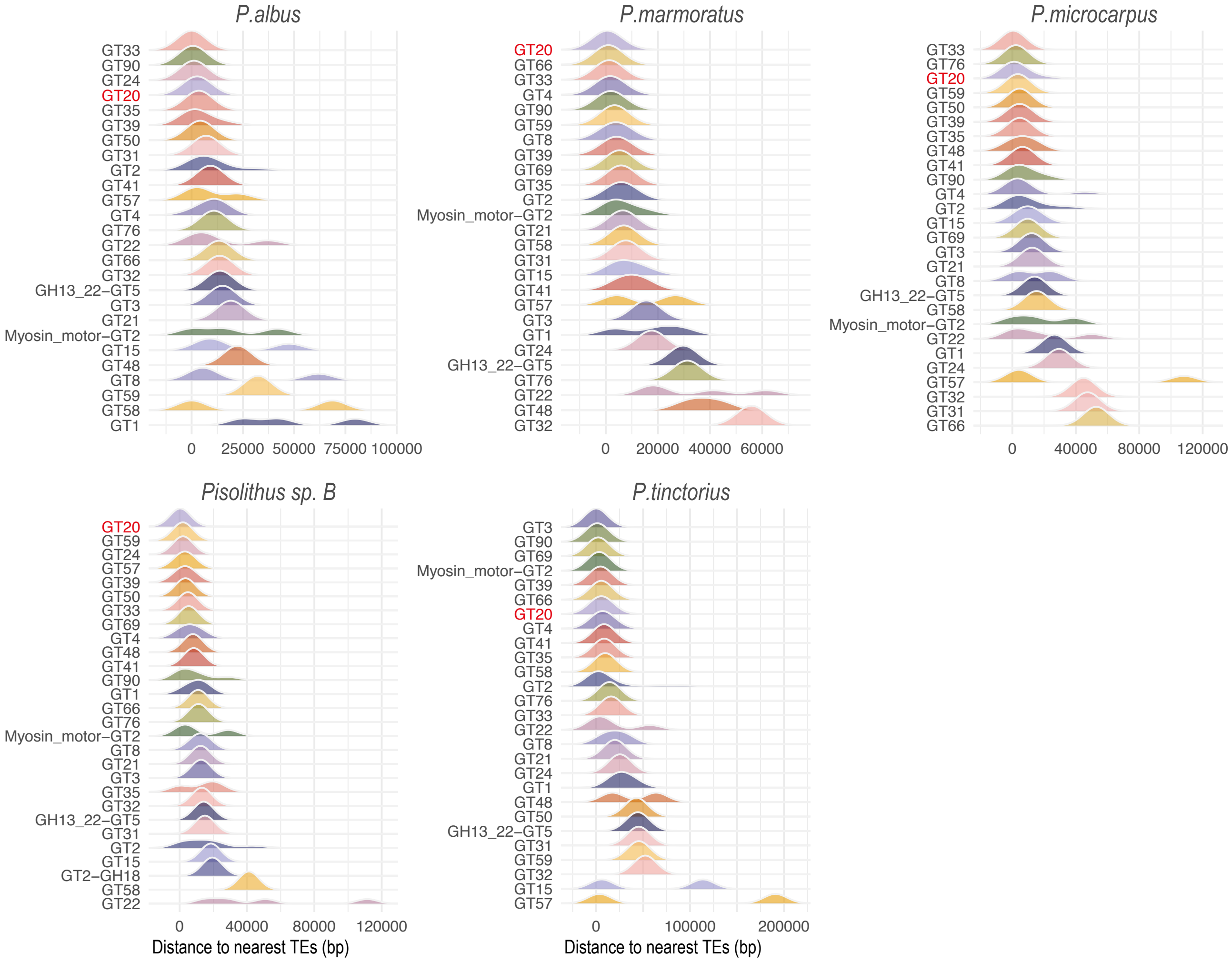

Figure S10: Density map of glycosyl hydrolase (GT) coding genes to the nearest transposable elements. The gene of interest, GT20 (in red) has a tendency to be close to TEs in five *Pisolithus* species.

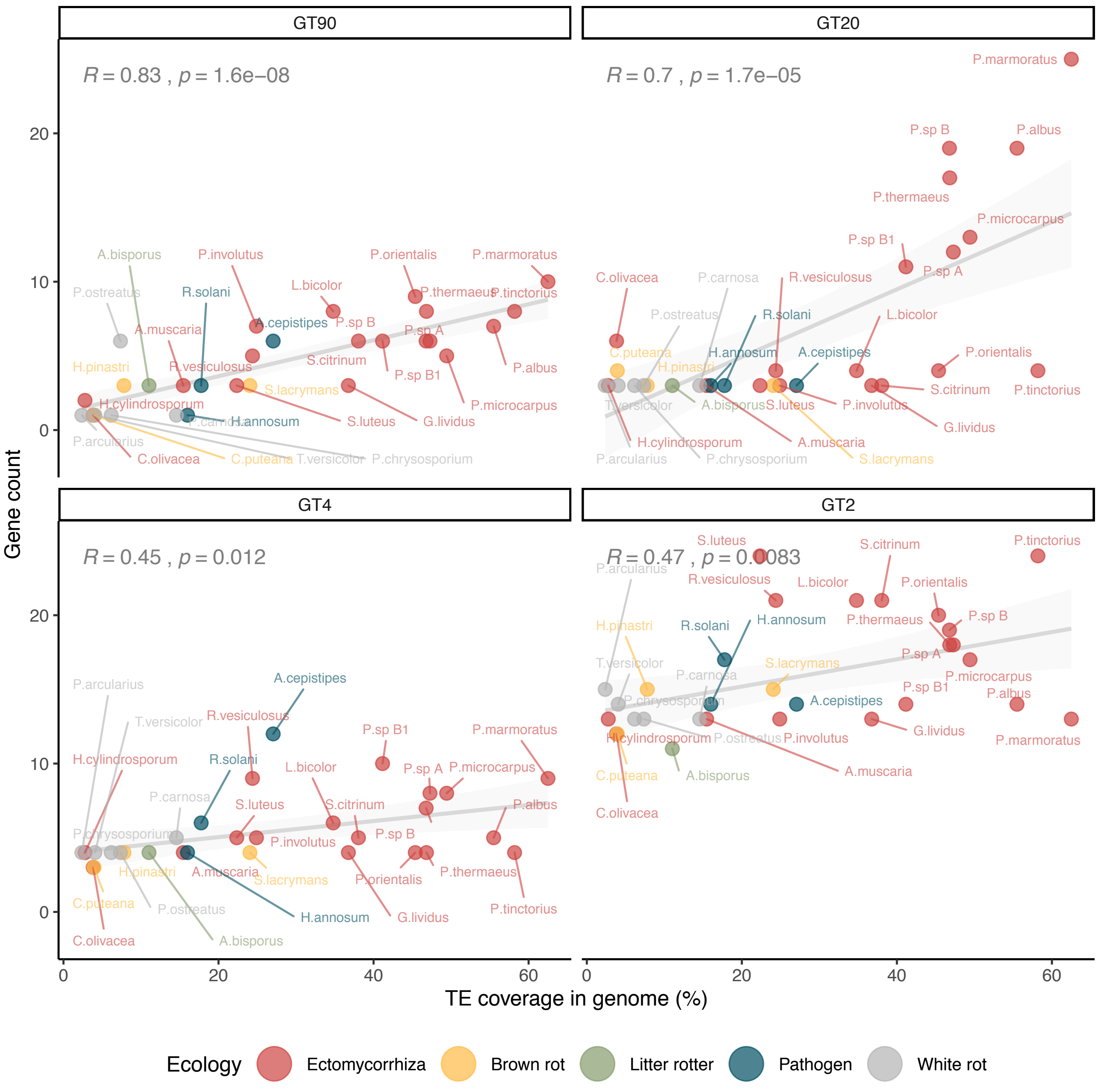

Figure S11. High correlation of the count of selected glycosyl transferases and repeat element content in the genomes. Fungal ecological groups are in color.

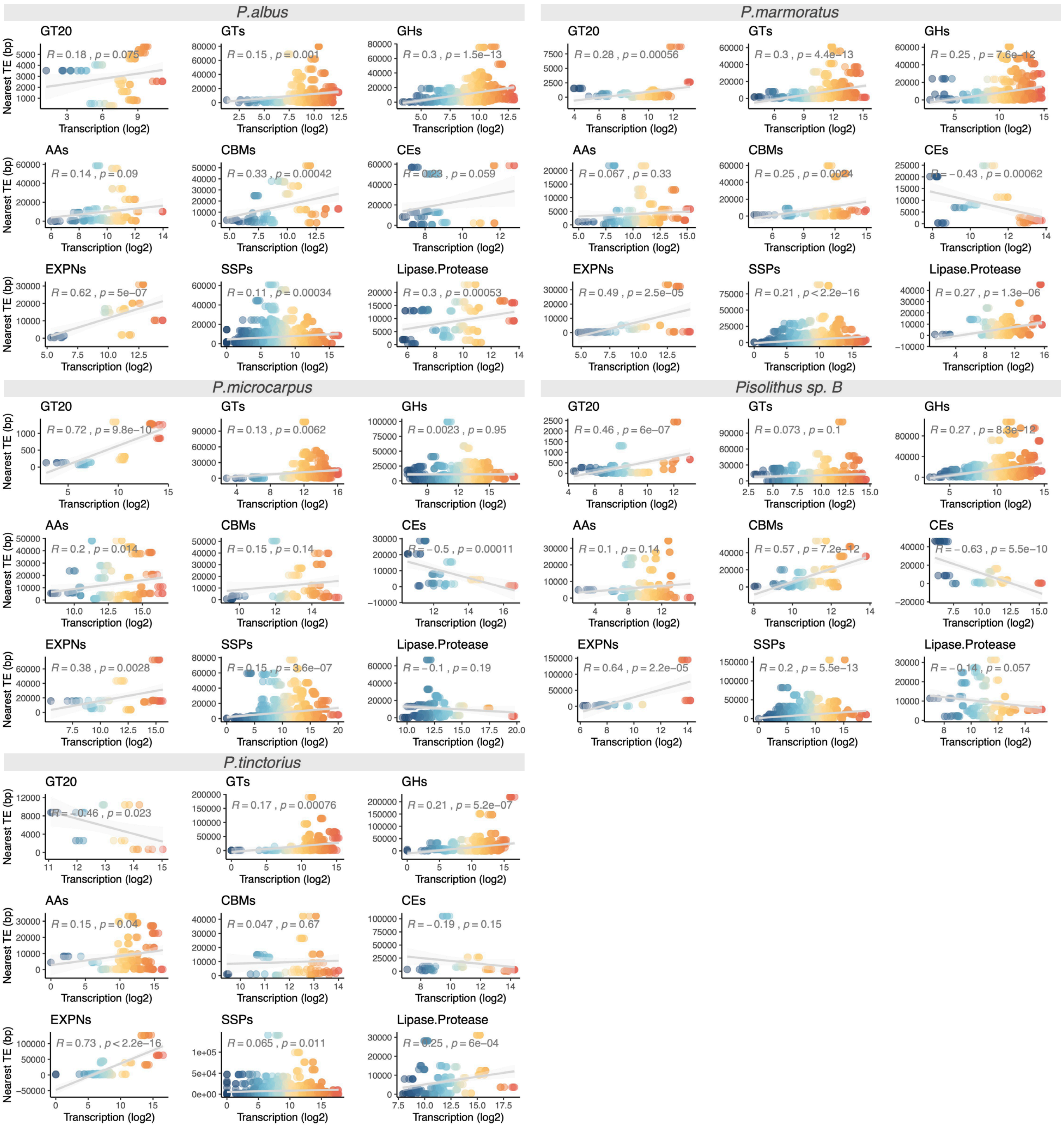

Figure S12. Correlations of gene-TE distance and transcription levels for various categories of genes in five *Pisolithus* fungi. Dots in color represent the transcription level. Correlations are indicated with Pearson correlation coefficient, p values, and linear regression lines.

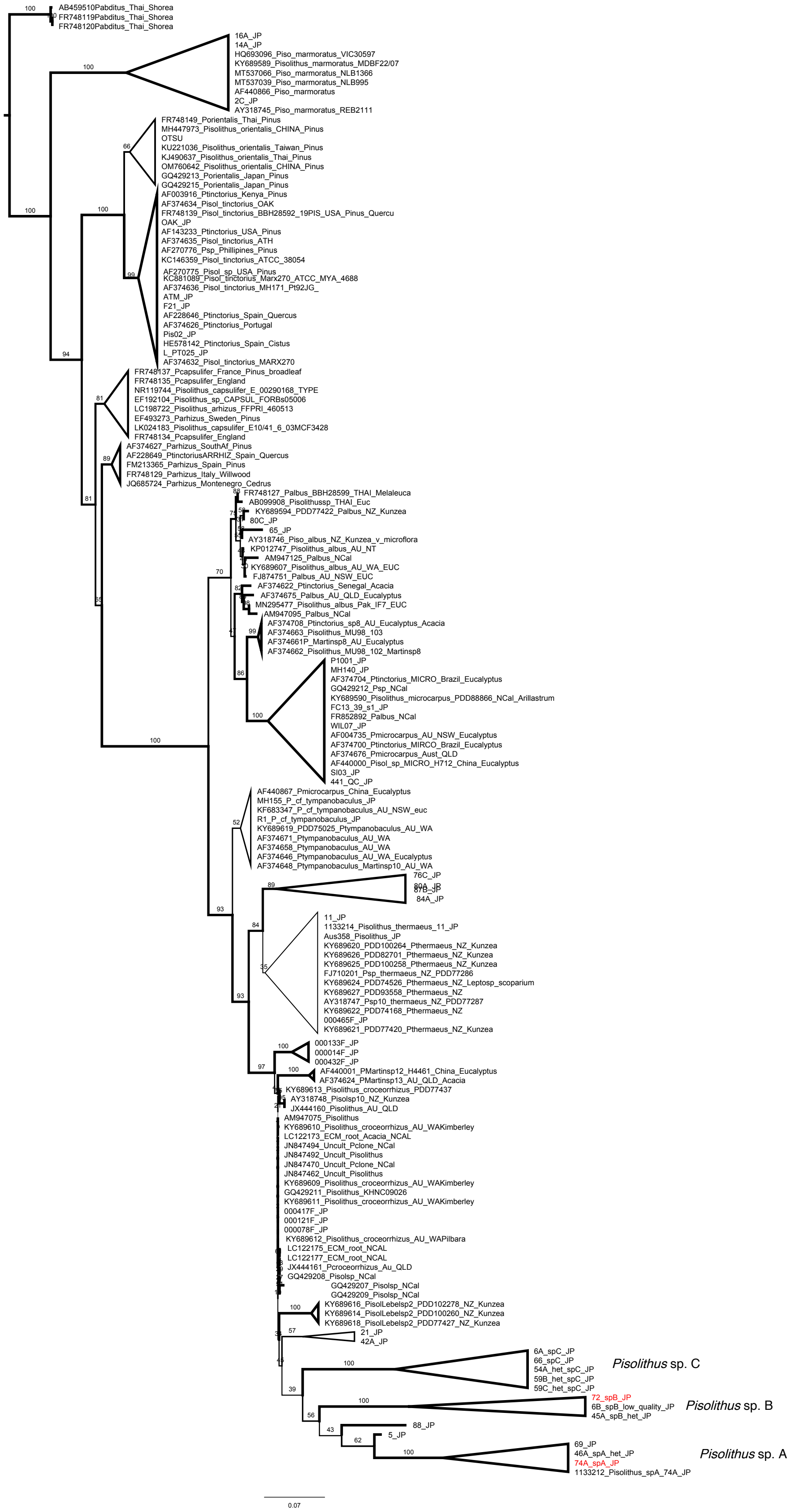

Figure S13. Maximum likelihood tree of fungal species based on the internal transcribed spacer regions. Clades are cartooned for species, to help clarify boundaries. Sequences generated for this project appended by 'JP', and those from the putative new species A and B used in this study are highlighted in red text. Bolded lines represent ML support > 70 %.
